# Multi-strain carriage and intrahost diversity of Staphylococcus aureus among Indigenous adults in the USA

**DOI:** 10.1099/mgen.0.001367

**Published:** 2025-03-14

**Authors:** Julia Webb, Eleonora Cella, Catherine G. Sutcliffe, Catherine Johnston, Sayf Al-Deen Hassouneh, Mohammad Jubair, Dennie Parker Riley, Carol Tso, Robert C. Weatherholtz, Laura L. Hammitt, Taj Azarian

**Affiliations:** 1 Burnett School of Biomedical Sciences, University of Central Florida, Orlando, FL, USA; 2 Center for Indigenous Health, Johns Hopkins Bloomberg School of Public Health, Baltimore, MD, USA

**Keywords:** carriage, genomics, Indigenous health, *Staphylococcus aureus*

## Abstract

*Staphylococcus aureus* (SA) is an opportunistic pathogen and human commensal that is frequently present in the upper respiratory tract, gastrointestinal tract and skin. While SA can cause diseases ranging from minor skin infections to life-threatening bacteraemia, it can also be carried asymptomatically. Indigenous individuals in the Southwest USA experience high rates of invasive SA disease. As carriage is the most significant risk factor for disease, understanding the dynamics of SA carriage, and in particular co-carriage of multiple strains, is important to develop strategies to prevent transmission in vulnerable communities. Here, we investigated SA co-carriage and intrahost evolution by sampling several colonies from multiple anatomical sites and whole-genome sequencing (WGS) on 310 SA isolates collected from 60 Indigenous adults participating in a cross-sectional carriage study. We assessed the richness and diversity of SA isolates *via* differences in multilocus sequence type, core-genome SNPs and genome content. Using WGS data, we identified 95 distinct SA intra-subject lineages (ISLs) among 60 participants; co-carriage was detected in 42% (25/60). Notably, two participants each carried four distinct SA ISLs. Variation in antibiotic resistance determinants among carried strains was identified among 42% (25/60) of participants. Lastly, we found unequal distribution of clonal complex by body site, suggesting that certain lineages may be adapted to specific anatomical sites. Together, these findings suggest that co-carriage may occur more frequently than previously appreciated and further our understanding of SA intrahost diversity during carriage, which has implications for surveillance activities and epidemiological investigations.

## Data Summary

The *Staphylococcus aureus* genome sequences are available from the National Center for Biotechnology Information Sequence Read Archive. The accession numbers are included in File S1 (available with the online version of this article). Participant data collected from Indigenous individuals are governed by the participating tribal nations. Data can be made available upon request (contact lhammitt@jhu.edu or csutcli1@jhu.edu), if it is consistent with the Institutional Review Board-approved protocol and if the disclosure is approved by the participating tribes.

**Impact Statement**

*Staphylococcus aureus* is a human commensal and pathogen that causes a wide range of infections, from mild skin and soft tissue infections to severe invasive diseases, including bacteraemia and pneumonia. Indigenous populations in the USA have experienced high rates of severe *S. aureus* disease; yet, little is known about rates of co-carriage of multiple *S. aureus* strains in these communities. To address this, we studied 60 asymptomatic *S. aureus* carriers recruited from Indigenous communities, sampling from multiple anatomical sites. From positive samples, we collected up to 6 *S. aureus* colonies and subsequently performed whole-genome sequencing on 310 isolates. Long-read sequencing was used to generate a closed intrahost reference genome for each participant, which significantly improved the resolution of within-host variation and enhanced our ability to identify co-carriage. Among carriers, we identified that 41.7% harboured more than one *S. aureus* intra-subject lineage. We also observed variability in genome content, including antibiotic resistance and virulence factors among co-carried strains. This multi-isolate genomic analysis overcomes previous limitations in sampling strategy and enhances our understanding of intrahost diversity during *S. aureus* carriage. Our findings suggest that co-carriage is more common than previously thought, which has implications for surveillance activities and epidemiological investigations.

## Introduction

*Staphylococcus aureus* (SA) is a Gram-positive opportunistic pathogen frequently carried asymptomatically in the gastrointestinal tract, upper respiratory system and skin. While SA can cause a variety of invasive and non-invasive diseases, estimates of asymptomatic carriage range from 20 to over 60% of the general population [1,2]. The most common site of carriage is the anterior nares, but there is an increasing focus on the oropharynx as an additional carriage site [3,7]. Carriage is thought to occur in three main patterns over time: persistent, intermittent or occasional and non-carriage [8]. Persistent carriers generally carry a single strain and have higher carriage density and greater risk of infection. Intermittent/occasional carriers may carry multiple strains at different time points or even concurrently [8].

SA carriage is one of the most significant risk factors for disease [9,10], and as such, understanding the dynamics of carriage is central to investigating transmission and developing effective prevention strategies. While carriage studies are abundant, those that employ sampling of multiple anatomical sites or perform molecular or genomic analysis of more than a single isolate from a participant are far fewer [11,13]. Therefore, instances of co-carriage are likely to be underestimated. Further, while typing methods including pulsed-field gel electrophoresis (PFGE), multilocus sequence typing (MLST) and *spa*-typing have historically been used to define the population structure of SA and to infer the genetic relatedness among strains [14,15], the increasing application of microbial genome sequencing has revealed their limited resolution. Through the application of pathogen genome sequencing, we now appreciate the breadth of intrahost microbial diversity that can exist during a single episode of carriage or disease [12,16, 17]. Yet, due to varying methodologies and definitions of a strain, the frequency of co-carriage remains unclear with prevalence estimates ranging from 9 to 79% (average 34%) [18,21]. The scarcity and vast range of available co-carriage estimates highlight the need for further studies and standardization of a co-carriage definition.

We recently completed a SA carriage study among Indigenous children and adults in the Southwest USA, where the rates of invasive disease are significantly higher than the general US population [22,23]. Using sampling of multiple upper respiratory anatomical sites and sequencing of a single SA isolate from each participant, the carriage prevalence of SA and methicillin-resistant *Staphylococcus aureus* (MRSA) were 20.7 and 1.7% among children <5 years, 30.2 and 2.8% among adults 18–64 years and 16.7 and 3.3% among adults ≥65 years, respectively [24]. Here, we expand on this work by sequencing multiple isolates from each anatomical site for each participant to determine the prevalence of co-carriage, characterize intrahost pathogen genetic diversity and explore the association between carried strains, anatomical site and strain–strain interactions.

## Methods

### Study population and sample collection

Sample collection and study population were described in detail by Cella *et al*. [24]. Briefly, the cross-sectional study included 288 Indigenous children (<5 years) and adults (≥18 years) living in the Navajo Nation and White Mountain Apache Tribal lands. Participants were recruited in 2017 *via* convenience sampling at well/routine healthcare visits and community events. Swab samples were collected from the anterior nares (AN) and nasopharynx (NP) of child participants and the AN, NP and oropharynx (OP) of adult participants. Isolation of SA from each swab was performed individually using BBL CHROMagar Staph aureus selective media plates (Becton Dickinson, Franklin Lakes, NJ). From positive plates, up to 6 SA colonies (henceforth ‘isolates’) were plated on a 6-zone blood agar plate. Each isolate was confirmed as SA using BBL Staphyloslide latex agglutination test (Becton Dickinson, Franklin Lakes, NJ) and then inoculated into 1 ml Bacto tryptic soy broth (TSB) (Becton Dickinson, Franklin Lakes, NJ) + 20% glycerol mix and stored at −80 °C.

In the primary study, 91 participants were found to be SA carriers, including 25 children <5 years of age. As the primary focus of this study was to assess intrahost SA diversity across multiple anatomical sites, we focused this analysis on adults (*n*=66) with samples collected from all three sites. Six adult participants had an insufficient number of sequenced isolates (<3) for meaningful analysis and were excluded; 60 participants were included in the final analysis.

### Bacterial gDNA isolation and whole-genome sequencing

Bacterial DNA extraction and whole-genome sequencing (WGS) were carried out as previously described [24]. Briefly, isolates were grown overnight in TSB and then treated with 0.1 mg ml^−1^ lysostaphin. Bacterial genomic DNA was extracted using the Qiagen DNeasy Blood and Tissue kit with 50 mg ml^−1^ lysozyme added to the lysis buffer. DNA quality and quantity were assessed using a Qubit 4 Fluorometer. Short-read sequencing libraries were constructed using the Nextera Flex and Illumina DNA Prep kits and sequenced using an Illumina MiSeq with 600-cycle v3 and 500-cycle v2 flow cells. Previously, one isolate per participant also underwent long-read sequencing using Oxford Nanopore Technologies MinION, allowing for the closure of most genomes. These high-quality draft assemblies also were included in the present study.

### Bioinformatics and phylogenetics

*De novo* assembly of sequencing reads was performed using Unicycler v0.5.0 [25], and assemblies were annotated using Prokka v1.14.5 [26]. The MLST of each isolate was determined using the tool MLST (https://github.com/tseemann/mlst) and the PubMLST database [27]. Antibiotic resistance and virulence factor presence were assessed using Abricate (https://github.com/tseemann/abricate) with databases ARG-ANNOT [28] and VFDB [29]. *De novo* assemblies were then used for pangenome analysis, which was carried out using Roary v3.13.0 [30]. SNP-sites v2.5.1 [31] was then used to extract polymorphic sites from the core-SNP alignment. IQ-TREE v1.6.12 [32] with the TVM+F+ASC+R2 substitution model [33,34] was used to create a maximum likelihood (ML) phylogeny. The phylogenetic tree was visualized and annotated using ggTree [35,37] run in RStudio v.4.1 [38]. Statistical analysis of clonal complex (CC) and MLST prevalence was performed using a Monte Carlo estimation of log odds ratio (https://github.com/sayfaldeen/BioinformaticsScripts/blob/main/MC-LOR-comp.py).

To assess intrahost diversity, an initial intrahost reference-based assembly was generated by mapping each participant’s short-read sequenced isolate to the respective high-quality hybrid-assembled genome using Snippy v4.6.0 (https://github.com/tseemann/snippy). These high-quality hybrid genome assemblies were constructed using 2×300 bp Illumina short-read data and long-read data generated using an Oxford Nanopore Technologies MinION, as previously described [24]. Pairwise SNP distances were calculated using snp-dists v0.8.2 for each intra-subject lineage (ISL) consensus alignment comprised of isolates belonging to the same MLST. We then examined the distribution of SNP distances for each ISL, considering the anatomic site of collection and spatial distribution of SNPs in the genome. In instances of co-carriage of distinct ISLs, for which we had not previously generated a closed genome through hybrid assembly, the closest matching publicly available reference genome was identified using the KmerFinder online tool (https://cge.food.dtu.dk/services/KmerFinder/). Once ISLs were defined, we plotted all pairwise SNP distances as well as the mean intrahost SNP distances for each one.

### Strain definitions

Distinct ISLs were delineated using MLST, and genetic distance was measured from the core genome. ISLs were defined initially as belonging to the same MLST. After reference-based mapping, as described above, and assessment of intrahost pairwise SNP distance, an empirically defined SNP cut-off of 100 was used to further delineate distinct ISLs, i.e. genomes diverging from the reference by more than the cut-off were considered a separate ISL indicative of a distinct carriage event. Therefore, co-carriage was defined as individuals carrying more than one ISL as defined by MLST type or SNP cutoff of 100 and mono-carriage as carrying only one ISL. Throughout the text, the term strain is used to broadly define groups of genetically distinct SA isolates, with strains collected from the same participant referred to as ISLs. The term sample is used to describe the swabs collected from a participant. Lastly, the terms isolate and genome refer to an individual bacterium and have no connotation of genetic relatedness or population structure.

### Co-carriage dynamics

We evaluated individual and household risk factors for co-carriage using log-binomial regression to estimate prevalence ratios (PR) and 95% confidence intervals as previously described [24]. Given the small sample size, a limited multivariable analysis was performed, and only age-adjusted PRs were presented. SAS software, version 9.4 of the SAS System for Windows (SAS Institute), was used for the risk factor analysis.

Next, we assessed discordance in antibiotic resistance (*mecA*, *blaZ*, *AadD*, *Aph3.III*, *Sat*4A, *Spc*, *erm*, *mphC* and *msrA*) and virulence factor (LukSF-PV, TSST, *etb*, enterotoxin, *can*, *chp*, *scn* and *vWbp*) determinants among co-carried strains, first considering co-carriage of MRSA and methicillin susceptible SA (MSSA). We then identified variations in antibiotic resistance and virulence profiles, focusing on the gain of determinants. For example, if an ISL was comprised of four isolates and one of them possessed *erm*, we considered that as discordance in the antibiotic profile. We chose this approach because genotypic identification of determinants is more prone to false negatives than false positives due to genome assembly errors. In instances when differences of multiple determinants were identified, we further investigated differences in plasmid and bacteriophage content.

Last, we examined the phylogenetic relationship between co-carried strains. We first visualized the ML phylogeny in a Circos plot with connections between co-carried isolates belonging to the same participant. Then, we sought to determine whether co-carried strains were more likely to be closely related phylogenetically as compared to other non-co-carried strains. After abstracting a pairwise distance matrix from the ML phylogeny, we plotted the distribution of pairwise distances among co-carried strains and non-co-carried strains separately. To statistically test whether the distances among co-carried and non-co-carried strains were significantly different, we conducted a permutation test by subsampling 200 pairs of isolates equally balanced between co-carried and non-co-carried strains for 1000 iterations. We then conducted a Mann-Whitney *U* test comparing the two distributions of mean ML distances to determine if they were the same and to obtain an empirical *P*-value. To assess the effect size, we calculated Cohen’s *d* and defined small effect size as *d*=0.2, medium effect size as *d*=0.5 and large effect size as *d*=0.8 [39].

## Results

### SA carriage

**Fig. 1.**
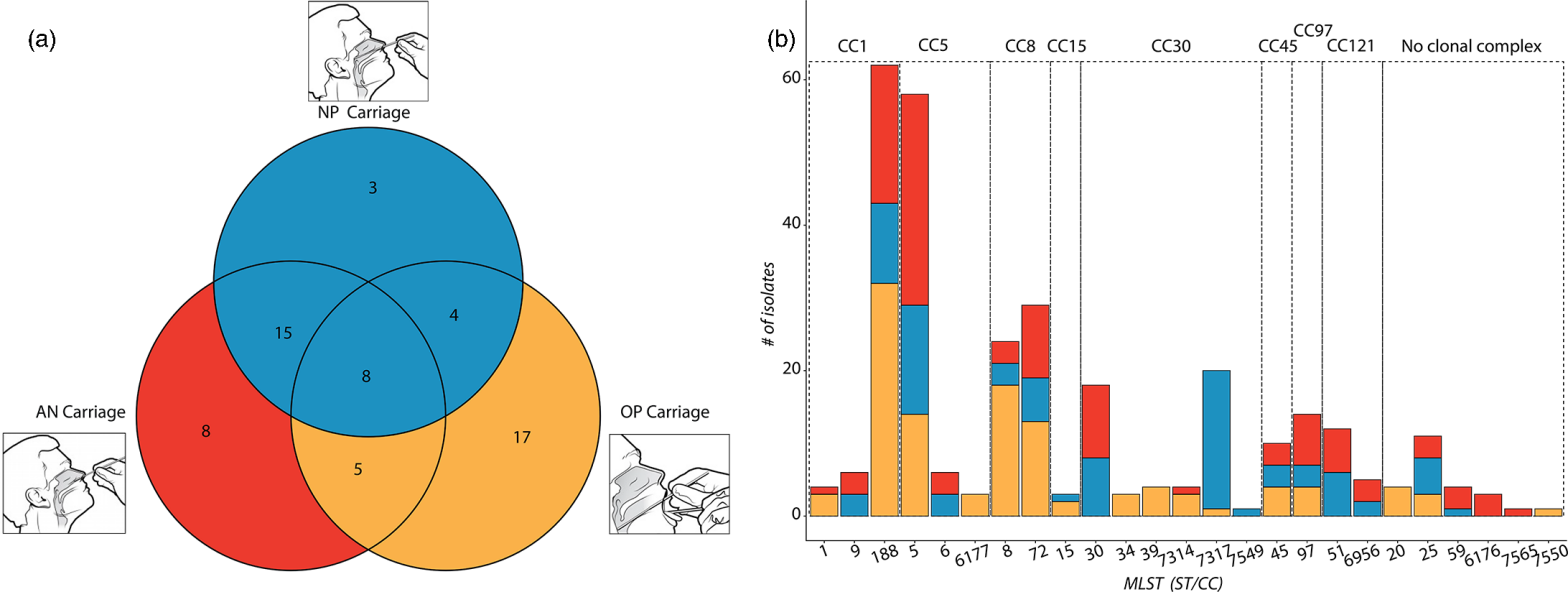
(a) SA carriage among Indigenous adults. Each of the three circles represents an anatomical site of carriage, and the counts within denote the number of participants with carriage at the given sites. (b) Lineage of isolates. The number of isolates belonging to each CC and each MLST within each clonal complex is shown. Each bar is divided to indicate the number of isolates taken from each anatomical site; OP (yellow), NP (blue) and AN (red).

Among the 60 adult participants, SA carriage positivity was similar by size: 36 (60.0%) in AN, 30 (50.0%) in NP and 34 (56.7%) in OP (Fig. 1a). Twenty-eight (46.7%) participants carried at only 1 anatomical site, 24 (40.0%) participants carried at 2 anatomical sites, and 8 (13.3%) participants carried at 3 anatomical sites. The most common carriage site was OP (60.7%) among one-site carriers and AN+NP (62.5%) among two-site carriers. Between 4 and 9 isolates were sequenced for each participant (mean=5.2, sd=1.6), for a total of 310 isolates across all participants (Table 1), including 108 (34.8%) from AN, 90 (29.0%) from NP and 112 (36.1%) from OP.

**Table 1.** SA isolates by anatomical site among Indigenous adults. Number of sequenced SA isolates and proportion of total from each anatomical site, by whether they were taken from a participant that carried SA at one, two or three anatomical sites

Carriers	AN isolates(% of total)	NP isolates(% of total)	OP isolates(% of total)	Total isolates(% of total)
One-site carriers	32 (10.3)	15 (4.8)	74 (23.9)	121 (39.0)
Two-site carriers	52 (16.8)	51 (16.5)	18 (5.8)	121 (39.0)
Three-site carriers	24 (7.7)	24 (7.7)	20 (6.5)	68 (21.9)
**Grand total**	**108** (34.8)	**90** (29.0)	**112** (36.1)	**310**

### Population structure

To classify isolates by their major lineages, we determined the MLST of each SA genome. This identified 25 unique sequence types (STs) belonging to 8 major CCs, led by CC1 (23.2%), CC5 (21.6%), CC8 (17.1%) and CC30 (16.1%) (Fig. 1b). The most prevalent CCs, CC1 and CC5, were both dominated by single MLSTs, ST188 and ST5, respectively. The distribution of CC by body site was significantly different. Notably, CC5 was disproportionately carried in the anterior nares, with 47.8% of CC5 isolates obtained from that anatomic site, CC30 was disproportionately carried in the nasopharynx, and CC1 and CC8 were more frequently isolated from the oropharynx (Table 2). Similarly, within CCs, some MLSTs were dominated by the isolates from specific anatomical sites. For example, 95.0% of ST7317 isolates were obtained from the nasopharynx. At the individual level, we observed the same pattern, with the exception of a slight shift in the prevalence of ST188 and ST5 (Fig. S1, available in the online Supplementary Material).

**Table 2.** CC prevalence comparison among body sites: AN, OP and NP Pairwise comparisons of carriage by anatomic site. Odd ratios and 95% highest posterior density (HPD) are given for each comparison, and significant values are bolded.

**Clonal complex**	Anterior nares (AN, *n*=108) vs.Other (*n*=202)		Nasopharynx (NP, *n*=90) vs.Other (*n*=220)		Oropharynx (OP, *n*=112) vs.Other (*n*=198)	
	Body site	Odds ratio (95% HPD)	Body site	Odds ratio(95% HPD)	Body site	Odds ratio(95% HPD)
CC1	AN (*n*=23)	0.87 (0.63–1.18)	NP (*n*=14)	0.59 (0.42–0.80)	OP (*n*=35)	**1.69 (1.29–2.41)**
	Other (*n*=49)	Ref	Other (*n*=58)	Ref	Other (*n*=37)	Ref
CC5	AN (*n*=32)	**1.68 (1.21–2.34)**	NP (*n*=18)	0.91 (0.66–1.24)	OP (*n*=17)	0.60 (0.42–0.83)
	Other (*n*=35)	Ref	Other (*n*=49)	Ref	Other (*n*=50)	Ref
CC8	AN (*n*=13)	0.59 (0.37–0.88)	NP (*n*=9)	0.51 (0.32–0.79)	OP (*n*=31)	**2.54 (1.76–3.72)**
	Other (*n*=40)	Ref	Other (*n*=44)	Ref	Other (*n*=22)	Ref
CC30	AN (*n*=11)	0.52 (0.32–0.78)	NP (*n*=28)	**3.15 (2.18–4.69)**	OP (*n*=11)	0.51 (0.33–0.76)
	Other (*n*=39)	Ref	Other (*n*=22)	Ref	Other (*n*=39)	Ref
CC45	AN (*n*=3)	0.78 (0.22–2.00)	NP (*n*=3)	1.11 (0.38–2.60)	OP (*n*=4)	1.22 (0.46–3.33)
	Other (*n*=7)	Ref	Other (*n*=7)	Ref	Other (*n*=6)	Ref
CC97	AN (*n*=7)	1.86 (0.88–4.67)	NP (*n*=3)	0.67 (0.25–1.44)	OP (*n*=4)	0.70 (0.29–1.71)
	Other (*n*=7)	Ref	Other (*n*=11)	Ref	Other (*n*=10)	Ref
CC121	AN (*n*=9)	**2.00 (1.09–4.75)**	NP (*n*=8)	**2.11 (1.13–5.25)**	–	
	Other (*n*=8)	Ref	Other (*n*=9)	Ref		

Assessing co-carriage, we found that 35 (58.3%) participants carried isolates belonging to a single MLST, 19 (31.7%) carried 2 MLST, and 6 (10.0%) carried 3 MLST, delineating 91 distinct ISLs among 60 individuals based on MLST and giving an overall proportion of co-carriage with multiple MLSTs of 41.7%(25/60).

### Intrahost diversity and co-carriage

**Fig. 2.**
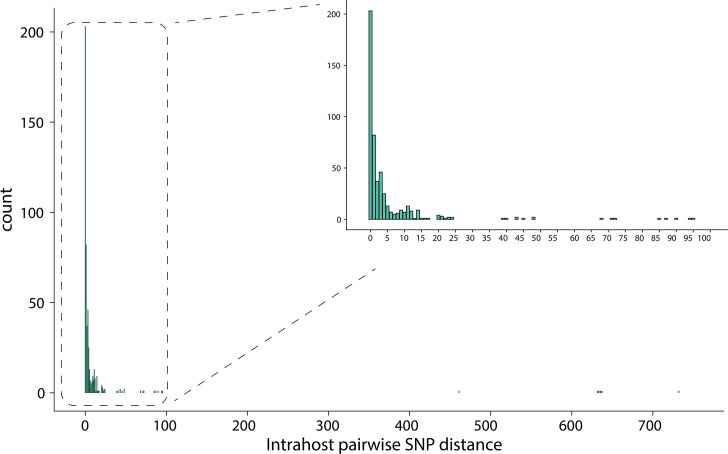
Pairwise SNP distances among intrahost individual SA ISLs with ≥2 isolates of the same MLST (*n*=78). An enlarged view of pairwise distances in the 0–100 SNP region of interest is shown on the top right of the figure. Several distances fall above the 39 SNP cutoff often used for inferring an epidemiological linkage.

To obtain greater resolution of intrahost diversity and co-carriage, we assessed pairwise SNP distances (Fig. 2). Of the 91 ISLs delineated by MLST, 78 had more than 1 isolate, allowing for pairwise comparison of genetic distance. Plotting intrahost pairwise SNP distances for these genomes showed that most resolved into genomically cohesive groups of genomes with distances ranging from 0 to 100 SNPs and some forming a long tail distribution from 450 to 750 SNPs (Fig. 2). As mean pairwise SNP distances are often used to describe population diversity, we additionally plotted the mean intrahost distances for each ISL, finding that most distances fell into the 0–50 SNPs range with a tail at 300–500 SNPs (mean=25.02, sd=95.05) (Fig. S2). Using an empirically defined cut-off of 100 SNPs, four participants carried isolates belonging to the same MLST that were further delineated into distinct ISLs. These genomes differed by intrahost SNP distances in the hundreds (mean=618.2) and did not demonstrate evidence of recent intrahost recombination events.

Taken together, using WGS data, we identified 95 distinct SA ISLs among 60 participants and an overall proportion of co-carriage of 25/60 (41.7%). If the six participants excluded for low numbers of isolates were single ISL carriers, the overall proportion of co-carriage would be 33.3% (25/66). Further, while WGS data did not identify additional co-carriers, it revealed that two participants carried four distinct ISLs each and two carried three each. Of note, using a more conservative cut-off of 39 SNPs to define ISLs, as previously suggested by Hall *et al.* [11], we would have delineated four additional co-carriers. Among co-carriage instances, 13/25 (52.0%) involved mixed ISLs within a specific anatomic site and 12 instances involved distinct ISLs compartmentalized at an anatomic site. In addition, 14, 18 and 20 instances of co-carriage involved OP, NP and AN sites, respectively. Notably, of the 18 instances of co-carriage involving the NP, 7 involved the same strain found at both AN and NP sites.

### Risk factors for co-carriage

Individual (Table S1) and household (Table S2) risk factors for co-carriage of distinct SA ISLs were assessed. Hospitalization in the last 6 months, gym use in the last 6 months and living in a household with seven or more people were significantly correlated with co-carriage in bivariable and age-adjusted analyses. While participation in team sports and activities in the last 6 months and having an average of more than two people per bedroom were associated with co-carriage in bivariable analysis, these results were no longer statistically significant after adjusting for age. The three participants with co-carriage who reported large household sizes also had recent healthcare exposure, indicating that multiple risk factors may contribute to high rates of co-carriage.

### Phylogenomics

**Fig. 3.**
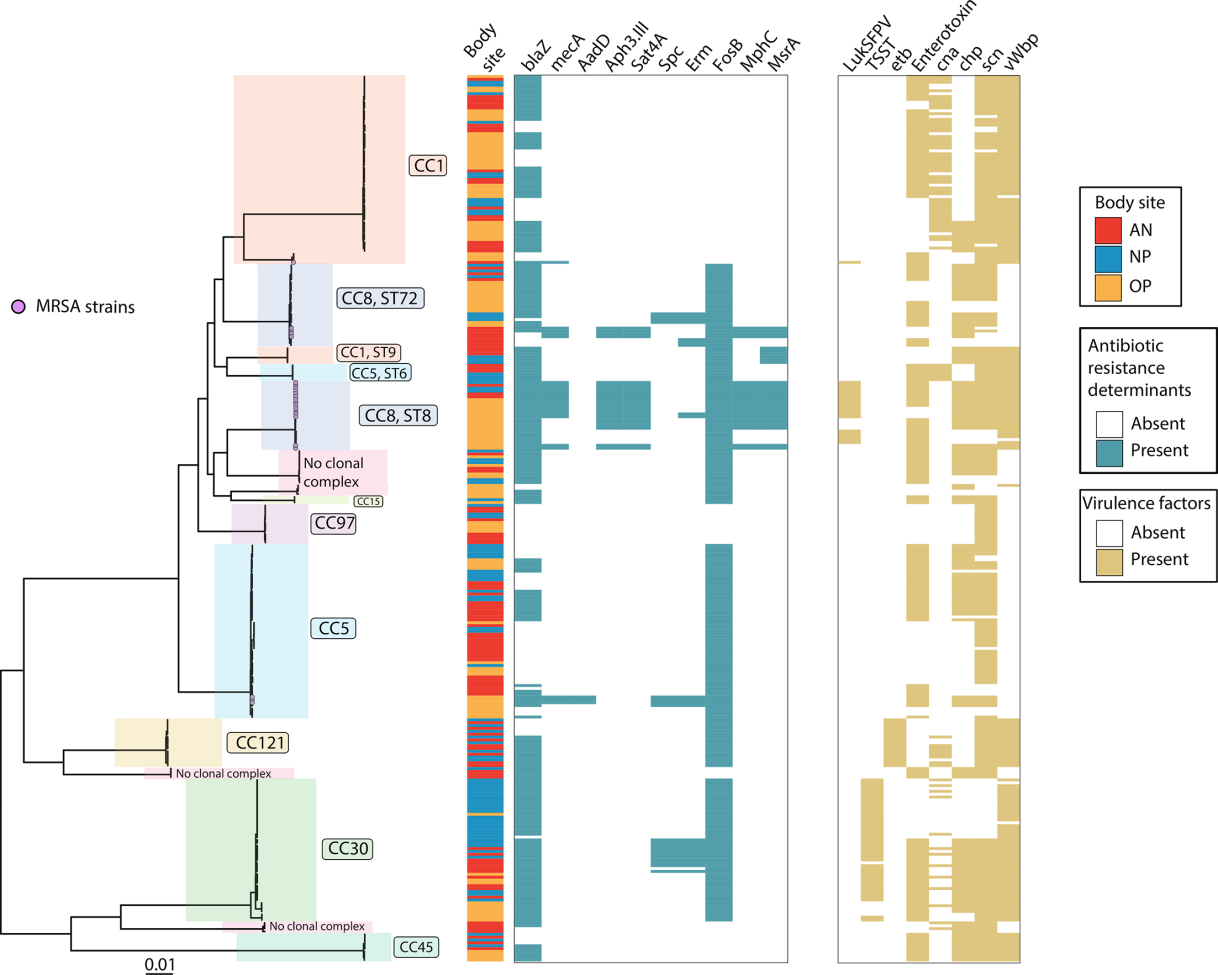
ML phylogeny of carried SA isolates and their associated traits. An ML phylogeny was generated from a core SNP alignment of all 310 isolates. Each tip corresponds to an isolate; a purple circle at the tip indicates the presence of *mecA* conferring methicillin resistance. Clades are organized by CC. To the right of the tree, the presence/absence matrices of antibiotic resistance determinants and virulence factors are shown. Antibiotic resistance determinants in order: *blaZ*, *mecA*, *AadD*, *Aph3.III*, *Sat*4A, *Spc*, *erm*, *mphC* and *msrA*. Virulence factors in order: LukSF-PV, TSST, *etb*, Enterotoxin, *can*, *chp*, *scn* and *vWbp*.

**Fig. 4.**
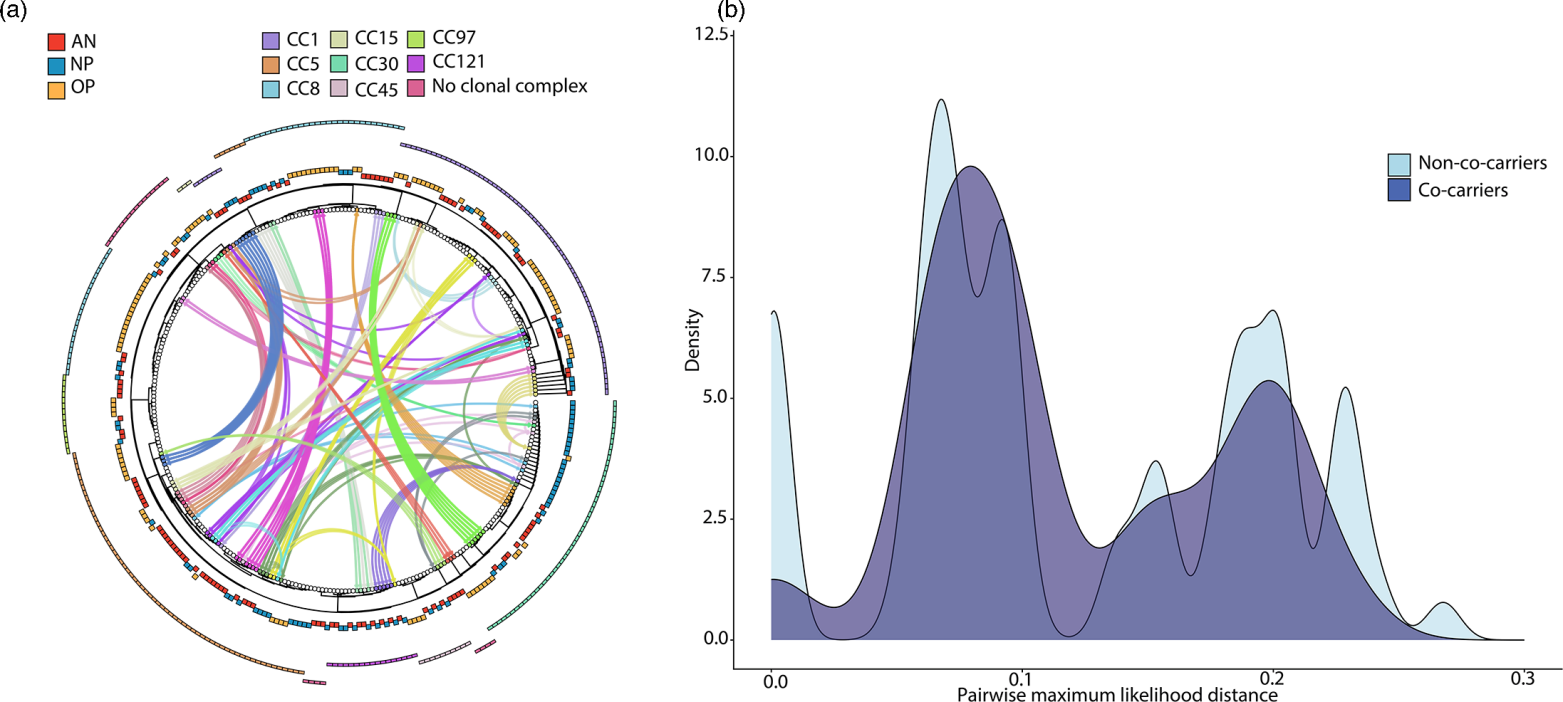
Circos plot of ML phylogeny and the relationship between co-carried SA strains. (a) An ML phylogeny was generated from a core SNP alignment of all 310 isolates. Each tip corresponds to an isolate. The first ring around the tree denotes anatomical site: AN, NP or OP. The outermost ring around the tree denotes CC. Inside the tree, connections are drawn between isolates that belong to the same participant; each participant is represented by a unique colour. (b) Density histogram plot comparing the pairwise ML genetic distance between co-carried strains from the same individual (dark blue) and the overall distribution of pairwise distances among all genomes from different individuals (non-co-carried strains, light blue).

To visualize the population structure and phylogenetic relationship between isolates, we constructed an ML phylogeny of all 310 genome assemblies and mapped the distribution of antibiotic resistance and virulence determinants (Fig. 3). The phylogeny shows that the majority of isolates cluster into eight major clades corresponding to CC. MRSA isolates, denoted by a star tip point, are almost exclusively found in CC8, which also predominantly harboured strains demonstrating aminoglycoside and macrolide resistance determinants and LukSF-PV encoding Panton–Valentine leucocidin. We found 25 instances of intrahost variation in antibiotic resistance determinants, accounting for 41.7% of the 60 participants studied. Of these, 20 instances were among individuals identified as co-carriers, while the other 5 instances were among mono-carriers. In three instances, we identified co-carriage of MRSA and MSSA strains belonging to two different MLSTs. The most common variation was observed with *blaZ* (18/25, 72.0%), which encodes *β*-lactamase and is associated with penicillin non-susceptibility, followed by *fosB* (10/25, 40.0%) and *erm* (8/25, 32.0%), which confer fosfomycin and macrolide resistance, respectively. Regarding variation in virulence determinants, we identified 38 instances of intrahost variation among 34 participants. Of these, 25 instances were among individuals identified as co-carriers, 9 instances were among mono-carriers, and 4 instances were among individuals with variation among co-carried strains (i.e. same MLSTs but a distinct ISL based on SNP distances) as well as within the ISL. Finally, to investigate the phylogenetic relationship among co-carried strains, we visualized the population structure illustrating links between strains isolated from the same participant and plotting the distribution of ML pairwise distances (Fig. 4). While the pairwise distances among co-carried strains were significantly less (i.e. more closely related) than the overall pairwise distances among non-co-carried strains, the effect size was small (Fig. S3).

## Discussion

Our investigation of co-carriage and intrahost diversity of SA among Indigenous adults in the Southwest USA, which involved sampling multiple respiratory anatomical sites and genomic analysis of multiple isolates, revealed a co-carriage prevalence of 41.7%. Notably, we resolved the degree of co-carriage (i.e. the number of distinct strains carried), finding six individuals carrying three distinct strains and two carrying four strains. Further, ISLs were found to vary in genome content, including antibiotic resistance and virulence determinants, which has considerable implications for carriage studies. Overall, our investigation of the intrahost diversity of SA carriage using a multi-site, multi-isolate, bacterial genome sequencing approach addressed previous limitations and updated our understanding of intrahost diversity during carriage.

Historically, SA carriage was generally thought to be clonal with only one ISL present [19]. Few studies have systematically explored the co-carriage of multiple SA strains. An early study of food handlers in 2003 revealed that 78.6% of participants (*n*=14) carried more than one strain, as defined by PFGE [18]. Subsequently, a comprehensive study of the clonality of SA carriage found that 14 out of 148 (9.4%) subjects carried isolates of different PFGE profiles, and 7 of these 14 subjects carried more than one strain as defined by MLST [19]. A paediatric study reported that 30.4% of carriers were colonized with more than one genotype as defined by multiple-locus variable-number tandem repeat fingerprinting [20], and Votintseva *et al.* found that over a 24-month period, 18% of subjects were co-colonized at some point by more than one strain as defined by *spa*-type [21].

More recently, the advent of bacterial genome sequencing demonstrated that previous typing methods lacked the resolution to delineate distinct strains within host lineages. Here, MLST was able to identify most co-carriers; however, the degree of co-carriage was only fully resolved through genomic sampling of multiple isolates from three anatomical sites. Our findings have significant implications for the use of genomic data to track pathogen transmission as well as understanding intra- and inter-host evolutionary rates. For example, previous studies have sought to establish a straightforward SNP-based cut-off to identify epidemiological linkage with upper-end limits set to 40 SNPs based on an estimated rate of 8 mutations per genome per year [11,40]. However, bioinformatics methods can vary considerably among studies, resulting in measurement differences in SNP distances. In particular, genome assembly methods and reference selection [41], in the case of reference-based assembly, can impact the ‘callable’ portions of the genome, thereby reducing resolution and artificially reducing distances. The use of an internal reference generated through the hybrid assembly of an intrahost strain increased our ability to assess microevolution at an unmatched level. Yet, it complicates our determination of ISLs as we have limited knowledge of the expected genomic variation when multiple upper respiratory sites are sampled. As a result, delineating ISLs that include strains with distances ranging from 40 to 100 SNPs remains speculative as these outliers could result from large transmission bottleneck size, extended durations of carriage, incomplete purifying selection, repeated self-inoculation from a reservoir body site or recurrent/ongoing household transmission [42]. The latter is consistent with the observed association between household size and co-carriage, further supporting the concept of long-term ongoing transmission of an endemic strain within the household. Subsequent longitudinal studies with repeated sampling of the household would allow us to resolve these dynamics. Nevertheless, our findings suggest that sampling several isolates from multiple body sites is important for transmission studies.

The oropharynx and anterior nares are the most frequently sampled sites for SA carriage [43,44]. While the nasopharynx is rarely sampled for SA [45] and is not thought to be a primary carriage site, we found that 50% of adult SA carriers had nasopharyngeal colonization. While it is possible that passage of a swab through the anterior nares during nasopharyngeal sampling could result in ‘contamination’ of the sample, the repeated identification of nasopharyngeal SA ISLs distinct from oropharyngeal and/or anterior nares samples co-carried in the same individual suggests that these findings represent true nasopharyngeal carriage. In addition, we found evidence of an association between specific clonal complexes and anatomic sites of isolation. CC5 was statistically significantly associated with isolation from the anterior nares, CC30 from the nasopharynx and CC1 and CC8 from the oropharynx. ST7317, a novel MLST belonging to CC30, was comprised almost entirely of isolates from the nasopharynx. While previous studies have observed an association of specific lineages (PFGE types of STs) with disease conditions such as ocular infections, atopic dermatitis and toxic shock syndrome [46,48], there are limited data on variation in strain type by anatomical site of carriage. A previous finding that PFGE-type USA300 (ST8) was associated with a high prevalence of oropharyngeal carriage in a New York prison population remains one of the few examples of preferential carriage by anatomic site and is consistent with our findings here [49]. Overall, our findings strongly suggest that lineages may be adapted for colonization of specific anatomical sites or that repeated exposure to new strains may compartmentalize ISLs across sites. These ideas require further exploration in future studies.

SA strains can vary considerably in genome content due to horizontal gene transfer [50], and the transfer of plasmids and bacteriophages has been documented during co-colonization [51]. We observed variations in antibiotic and virulence determinants throughout the phylogeny of co-carried strains. Assessing intrahost differences in more detail, we found that an appreciable proportion of individuals with both co- and mono-carriage had clinically relevant differences in virulence and antibiotic resistance determinants, even in instances where the individual ISLs resided in different anatomical sites. Most notable were instances of co-carriage of MRSA and MSSA strains, macrolide resistance conferred by *erm* and virulence genes for exfoliative toxin (*etb/eta*) and toxic-shock syndrome. Indeed, co-carriage provides an ideal setting for the exchange of mobile genetic elements. This finding further supports the need to sample multiple body sites in epidemiologic studies or clinical MRSA surveillance, as sampling a single anatomical site may miss MRSA carriage.

Further, little is known about intraspecies interactions during co-carriage. Co-carried strains compete for resources while simultaneously evading the host immune system and other colonizing species. Previously, competitive exclusion between strains was thought to limit the incidence of co-carriage [52]. For example, carriage of MSSA at a single anatomical site was found to be protective for MRSA acquisition [53]. As such, we posited that co-carried strains would be genomically divergent, since this would potentially maximize the antigenic divergence, thereby avoiding any strain-specific immunity to superantigens or capsular polysaccharides [54]. However, we observed no overt association between co-carried strains based on genetic divergence, as illustrated by the complexity in the Circos plot and distribution of pairwise SNP distances. Notwithstanding, an increased tolerance of a strain for co-colonization could be an evolutionary advantage as it would provide an opportunity for onward transmission as well as acquisition of mobile elements. Further, niche expansion through the ability to inhabit multiple respiratory sites would avoid direct competition for binding to epithelial cells and would further explain why we frequently observed compartmentalization of distinct ISLs across anatomical sites. A full accounting of genome content variation between mono- and co-carried strains as well as phenotypic and competition experiments may further elucidate the existence of preferred strain pairs and how these dynamics relate to strain prevalence and carriage duration.

Crowding, participation in team sports and healthcare contact are previously recognized risk factors for community-associated SA carriage and disease [55]. Therefore, one might expect these same risk factors to increase the likelihood of co-carriage. Indeed, we observed healthcare, household and community risk factors associated with the carriage of multiple SA strains. In our previous analysis, several household risk factors were associated with the carriage, including the history of SA infection among household members and household members sharing a bed or using a gym or locker room [24]. Consistent with these previous findings, the number of people in the household and the number of people per bedroom, both proxies for crowding, and healthcare exposure were found to be significantly associated with increased likelihood of co-carriage. As household size increases, multiple individual risk factors are compounded, likely leading to ongoing transmission of ‘endemic’ household strains and increasing the potential for introduction of new strains acquired from outside the household (e.g. gym or hospital). Together, this underscores the significance of household transmission of SA, as has been reported [56,57], and suggests an opportunity for interventions such as decolonization, enhanced environmental cleaning or education campaigns.

Our study was not without limitations; primarily, the inclusion only of adults leaves intrahost SA carriage in children yet to be explored. As children are an important reservoir for respiratory pathogens and contribute to community transmission [58], future study is merited. The small sample size limited our ability to fully explore the individual and household risk factors associated with co-carriage. Moreover, this study was cross-sectional, assessing samples taken only at a single timepoint for each participant. Additionally, despite the number of isolates per participant analysed here, there remains the potential underestimation of genetic diversity. Detecting variants at lower frequencies with high statistical confidence requires sampling more colonies than is currently feasible. Finally, standard culture-based detection methods may have led to false negatives. Despite these limitations, the lack of alternative high-throughput methods means this approach remains valuable. Future work should include longitudinal sampling with increased sample size, to explore the intrahost dynamics of SA carriage over time and the impact of co-carriage on the duration of carriage.

Overall, this work offers a high-resolution analysis of intrahost diversity among SA carriers. We found that an appreciable proportion of carriers were colonized by more than one MLST. In particular, our novel use of hybrid genome sequencing to generate an intrahost reference allowed us to assess intrahost evolution at a previously unexplored level of resolution. Together, these findings indicate that the diversity of SA within carriers is greater than previously appreciated. As accurate identification and characterization of SA carriage is central to surveillance activities and genomic epidemiology studies, we show the importance of considering intrahost diversity including co-carriage and incorporating these methods into future work.

## supplementary material

10.1099/mgen.0.001367 Uncited Supplementary Material 1.

## Abbreviations

AN: anterior nares
CC: clonal complex
HPD: highest posterior density
ISL: intra-subject lineage
ML: maximum likelihood
MLST: multilocus sequence type
MRSA: methicillin-resistant *Staphylococcus aureus*
MSSA: methicillin susceptible SA
NP: nasopharynx
OP: oropharynx
PFGE: pulsed-field gel electrophoresis
PR: prevalence ratios
SA: *Staphylococcus aureus*
SNP: single nucleotide polymorphism
ST: sequence type
TSB: tryptic soy broth
WGS: whole-genome sequencing

## References

[1] Gorwitz RJ, Kruszon-Moran D, McAllister SK, McQuillan G, McDougal LK (2008). Changes in the prevalence of nasal colonization with *Staphylococcus aureus* in the United States, 2001-2004. J Infect Dis.

[2] Russakoff B, Wood C, Lininger MR, Barger SD, Trotter RT (2023). A quantitative assessment of *Staphylococcus aureus* community carriage in Yuma, Arizona. J Infect Dis.

[3] Mertz D, Frei R, Periat N, Zimmerli M, Battegay M (2009). Exclusive *Staphylococcus aureus* throat carriage: at-risk populations. Arch Intern Med.

[4] Mertz D, Frei R, Jaussi B, Tietz A, Stebler C (2007). Throat swabs are necessary to reliably detect carriers of *Staphylococcus aureus*. Clin Infect Dis.

[5] Nilsson P, Ripa T (2006). *Staphylococcus aureus* throat colonization is more frequent than colonization in the anterior nares. J Clin Microbiol.

[6] Widmer AF, Mertz D, Frei R (2008). Necessity of screening of both the nose and the throat to detect methicillin-resistant *Staphylococcus aureus* colonization in patients upon admission to an intensive care unit. J Clin Microbiol.

[7] Ide L, Lootens J, Thibo P (2009). The nose is not the only relevant MRSA screening site. Clin Microbiol Infect.

[8] Kluytmans J, van Belkum A, Verbrugh H (1997). Nasal carriage of *Staphylococcus aureus*: epidemiology, underlying mechanisms, and associated risks. Clinical microbiology reviews.

[9] Fritz SA, Epplin EK, Garbutt J, Storch GA, Kaplan SL (2009). Skin infection in children colonized with community-associated methicillin-resistant *Staphylococcus aureus*. J Infect.

[10] Smyth DS, Kafer JM, Wasserman GA, Velickovic L, Mathema B (2012). Nasal carriage as a source of agr-defective *Staphylococcus aureus* bacteremia. J Infect Dis.

[11] Hall MD, Holden MT, Srisomang P, Mahavanakul W, Wuthiekanun V (2019). Improved characterisation of MRSA transmission using within-host bacterial sequence diversity. Elife.

[12] Golubchik T, Batty EM, Miller RR, Farr H, Young BC (2013). Within-host evolution of *Staphylococcus aureus* during asymptomatic carriage. PLoS One.

[13] Harkins CP, Pettigrew KA, Oravcová K, Gardner J, Hearn RMR (2018). The microevolution and epidemiology of *Staphylococcus aureus* colonization during atopic eczema disease flare. J Invest Dermatol.

[14] Tenover FC, Arbeit R, Archer G, Biddle J, Byrne S (1994). Comparison of traditional and molecular methods of typing isolates of *Staphylococcus aureus*. J Clin Microbiol.

[15] David MZ, Taylor A, Lynfield R, Boxrud DJ, Short G (2013). Comparing pulsed-field gel electrophoresis with multilocus sequence typing, spa typing, staphylococcal cassette chromosome mec (SCCmec) typing, and PCR for panton-valentine leukocidin, arcA, and opp3 in methicillin-resistant *Staphylococcus aureus* isolates at a U.S. Medical Center. J Clin Microbiol.

[16] Price JR, Golubchik T, Cole K, Wilson DJ, Crook DW (2014). Whole-genome sequencing shows that patient-to-patient transmission rarely accounts for acquisition of *Staphylococcus aureus* in an Intensive Care Unit. Clin Infect Dis.

[17] Long SW, Beres SB, Olsen RJ, Musser JM (2014). Absence of patient-to-patient intrahospital transmission of *Staphylococcus aureus* as determined by whole-genome sequencing. mBio.

[18] Acco M, Ferreira FS, Henriques JAP, Tondo EC (2003). Identification of multiple strains of *Staphylococcus aureus* colonizing nasal mucosa of food handlers. Food Microbiol.

[19] Cespedes C, Said-Salim B, Miller M, Lo S-H, Kreiswirth BN (2005). The clonality of *Staphylococcus aureus* nasal carriage. J Infect Dis.

[20] Mongkolrattanothai K, Gray BM, Mankin P, Stanfill AB, Pearl RH (2011). Simultaneous carriage of multiple genotypes of *Staphylococcus aureus* in children. J Med Microbiol.

[21] Votintseva AA, Miller RR, Fung R, Knox K, Godwin H (2014). Multiple-strain colonization in nasal carriers of *Staphylococcus aureus*. J Clin Microbiol.

[22] Sutcliffe CG, Grant LR, Reid A, Douglass GK, Weatherholtz RC (2019). The burden of *Staphylococcus aureus* among Native Americans on the Navajo Nation. PLoS One.

[23] Sutcliffe CG, Grant LR, Reid A, Douglass G, Brown LB (2020). High burden of *Staphylococcus aureus* among Native American individuals on the White Mountain Apache Tribal lands. Open Forum Infect Dis.

[24] Cella E, Sutcliffe CG, Tso C, Paul E, Ritchie N (2022). Carriage prevalence and genomic epidemiology of *Staphylococcus aureus* among Native American children and adults in the Southwestern USA. Microb Genom.

[25] Wick RR, Judd LM, Gorrie CL, Holt KE (2017). Unicycler: Resolving bacterial genome assemblies from short and long sequencing reads. PLoS Comput Biol.

[26] Seemann T (2014). Prokka: rapid prokaryotic genome annotation. Bioinformatics.

[27] Jolley KA, Bray JE, Maiden MCJ (2018). Open-access bacterial population genomics: BIGSdb software, the PubMLST.org website and their applications. Wellcome Open Res.

[28] Gupta SK, Padmanabhan BR, Diene SM, Lopez-Rojas R, Kempf M (2014). ARG-ANNOT, a new bioinformatic tool to discover antibiotic resistance genes in bacterial genomes. Antimicrob Agents Chemother.

[29] Chen L, Zheng D, Liu B, Yang J, Jin Q (2016). VFDB 2016: hierarchical and refined dataset for big data analysis—10 years on. Nucleic Acids Res.

[30] Page AJ, Cummins CA, Hunt M, Wong VK, Reuter S (2015). Roary: rapid large-scale prokaryote pan genome analysis. Bioinformatics.

[31] Page AJ, Taylor B, Delaney AJ, Soares J, Seemann T (2016). *SNP-sites*: rapid efficient extraction of SNPs from multi-FASTA alignments. Microb Genom.

[32] Minh BQ, Schmidt HA, Chernomor O, Schrempf D, Woodhams MD (2020). IQ-TREE 2: New models and efficient methods for phylogenetic inference in the genomic era. Mol Biol Evol.

[33] Kalyaanamoorthy S, Minh BQ, Wong TKF, von Haeseler A, Jermiin LS (2017). ModelFinder: fast model selection for accurate phylogenetic estimates. Nat Methods.

[34] Hoang DT, Chernomor O, von Haeseler A, Minh BQ, Vinh LS (2018). UFBoot2: Improving the ultrafast bootstrap approximation. Mol Biol Evol.

[35] Yu G, Lam TTY, Zhu H, Guan Y (2018). Two methods for mapping and visualizing associated data on phylogeny using ggtree. Mol Biol Evol.

[36] Yu G, Smith DK, Zhu H, Guan Y, Lam TTY (2017). Ggtree: an r package for visualization and annotation of phylogenetic trees with their covariates and other associated data. Methods Ecol Evol.

[37] Yu G (2020). Using ggtree to Visualize Data on Tree-Like Structures. Curr Protoc Bioinformatics.

[38] RStudio Team (2020). http://www.rstudio.com/.

[39] Cohen J (1992). A power primer. Psychol Bull.

[40] Young BC, Golubchik T, Batty EM, Fung R, Larner-Svensson H (2012). Evolutionary dynamics of *Staphylococcus aureus* during progression from carriage to disease. Proc Natl Acad Sci U S A.

[41] Valiente-Mullor C, Beamud B, Ansari I, Francés-Cuesta C, García-González N (2021). One is not enough: On the effects of reference genome for the mapping and subsequent analyses of short-reads. PLoS Comput Biol.

[42] Didelot X, Walker AS, Peto TE, Crook DW, Wilson DJ (2016). Within-host evolution of bacterial pathogens. Nat Rev Microbiol.

[43] Wertheim HFL, Melles DC, Vos MC, van Leeuwen W, van Belkum A (2005). The role of nasal carriage in *Staphylococcus aureus* infections. Lancet Infect Dis.

[44] McKinnell JA, Huang SS, Eells SJ, Cui E, Miller LG (2013). Quantifying the impact of extranasal testing of body sites for methicillin-resistant *Staphylococcus aureus* colonization at the time of hospital or intensive care unit admission. Infect Control Hosp Epidemiol.

[45] Bogaert D, van Belkum A, Sluijter M, Luijendijk A, de Groot R (2004). Colonisation by *Streptococcus pneumoniae* and *Staphylococcus aureus* in healthy children. Lancet.

[46] Parsonnet J, Hansmann MA, Jones MB, Ohtagaki K, Davis CC (2008). Prevalence of toxic shock syndrome toxin 1 (TSST-1)-producing strains of *Staphylococcus aureus* and antibody to TSST-1 among healthy japanese women. J Clin Microbiol.

[47] Benito D, Aspiroz C, Gilaberte Y, Sanmartín R, Hernández-Martin Á (2016). Genetic lineages and antimicrobial resistance genotypes in *Staphylococcus aureus* from children with atopic dermatitis: detection of clonal complexes CC1, CC97 and CC398. J Chemother.

[48] Wurster JI, Bispo PJM, Van Tyne D, Cadorette JJ, Boody R (2018). *Staphylococcus aureus* from ocular and otolaryngology infections are frequently resistant to clinically important antibiotics and are associated with lineages of community and hospital origins. PLoS One.

[49] Lee CJ, Sankaran S, Mukherjee DV, Apa ZL, Hafer CA (2011). *Staphylococcus aureus* oropharyngeal carriage in a prison population. Clin Infect Dis.

[50] Lindsay JA (2014). *Staphylococcus aureus* genomics and the impact of horizontal gene transfer. Int J Med Microbiol.

[51] McCarthy AJ, Loeffler A, Witney AA, Gould KA, Lloyd DH (2014). Extensive horizontal gene transfer during *Staphylococcus aureus* co-colonization in vivo. Genome Biol Evol.

[52] Aly R, Maibach HI, Shinefield HR, Mandel A, Strauss WG (1974). Bacterial interference among strains of *Staphylococcus aureus* in man. J Infect Dis.

[53] Dall’Antonia M, Coen PG, Wilks M, Whiley A, Millar M (2005). Competition between methicillin-sensitive and -resistant *Staphylococcus aureus* in the anterior nares. J Hosp Infect.

[54] Holtfreter S, Roschack K, Eichler P, Eske K, Holtfreter B (2006). *Staphylococcus aureus* carriers neutralize superantigens by antibodies specific for their colonizing strain: A potential explanation for their improved prognosis in severe sepsis. J Infect Dis.

[55] Salgado CD, Farr BM, Calfee DP (2003). Community-acquired methicillin-resistant *Staphylococcus aureus*: a meta-analysis of prevalence and risk factors. Clin Infect Dis.

[56] Alam MT, Read TD, Petit RA, Boyle-Vavra S, Miller LG (2015). Transmission and microevolution of USA300 MRSA in U.S. households: evidence from whole-genome sequencing. mBio.

[57] Uhlemann AC, Kennedy AD, Martens C, Porcella SF, Deleo FR (2012). Toward an understanding of the evolution of *Staphylococcus aureus* strain USA300 during colonization in community households. Genome Biol Evol.

[58] Esposito S, Terranova L, Zampiero A, Ierardi V, Rios WP (2014). Oropharyngeal and nasal *Staphylococcus aureus* carriage by healthy children. BMC Infect Dis.

