# Supplementary material for: Multi-strain carriage and intrahost diversity of Staphylococcus aureus among Indigenous adults in the USA
Source: Microb Genom. 2025 Mar 14;11(3):001367. doi: 10.1099/mgen.0.001367 (PMC11909137; doi:10.1099/mgen.0.001367)
Supplement: Uncited Supplementary Material 1. [file mgen-11-01367-s001.pdf]

Supplemental Material

Multi-strain carriage and intrahost diversity of *Staphylococcus aureus* among Indigenous adults in the US

Julia Webb, Eleonora Cella, Catherine Sutcliffe, Catherine Johnston, Sayf Al-Deen Hassouneh, Mohammad Jubair, Dennie Parker Riley, Carol Tso, Robert C. Weatherholtz, Laura L. Hammitt, Taj Azarian

Supplemental Figures

Supplemental Figure 1. Distribution of carried lineages by multi-locus sequence type (MLST) and clonal complex (CC).

Supplemental Figure 2. Mean pairwise SNP-distances among intrahost individual SA ISLs with ≥2 isolates of the same MLST (n=78).

Supplemental Figure 3. Results of permutation test assessing the maximum likelihood (ML) distances among co-carried from the same individual and non-co-carried strains from all individuals.

Supplemental Tables

Supplemental Table 1. Individual characteristics of study participants and correlates of co-carriage among Indigenous adults in the Southwest US in 2017.

Supplemental Table 2. Household characteristics of study participants and correlates of co-carriage among Indigenous adults in the Southwest US in 2017.

Supplemental Files

Supplemental File 1. Accession numbers

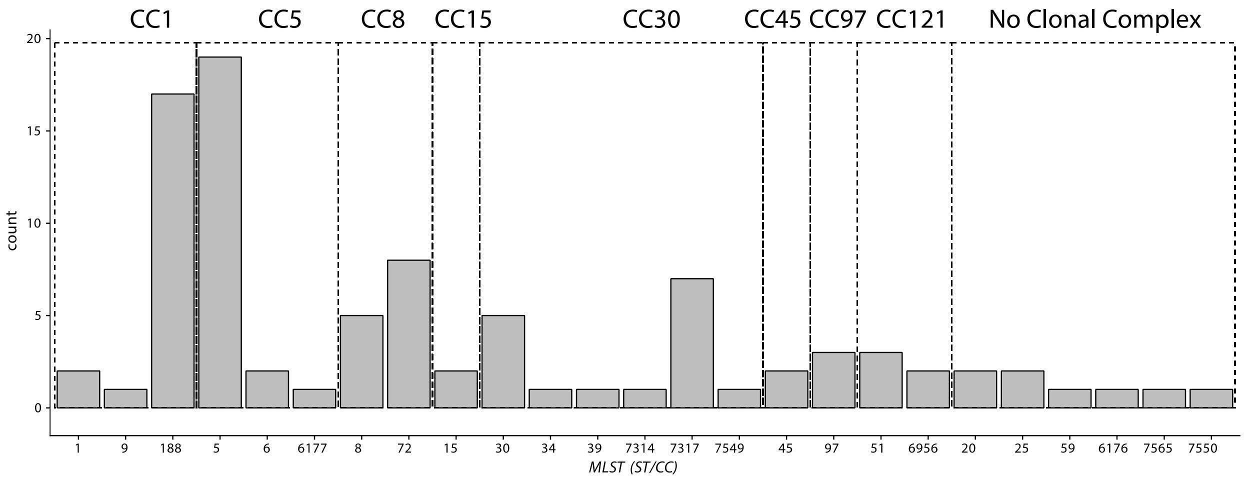

Supplemental Figure 1. Distribution of carried lineages by multi-locus sequence type (MLST) and clonal complex (CC). *The bar graph is stratified by the count of MLST. In total, 91 of MLSTs comprised of 310 isolates from 60 carriers are represented in the plot.*

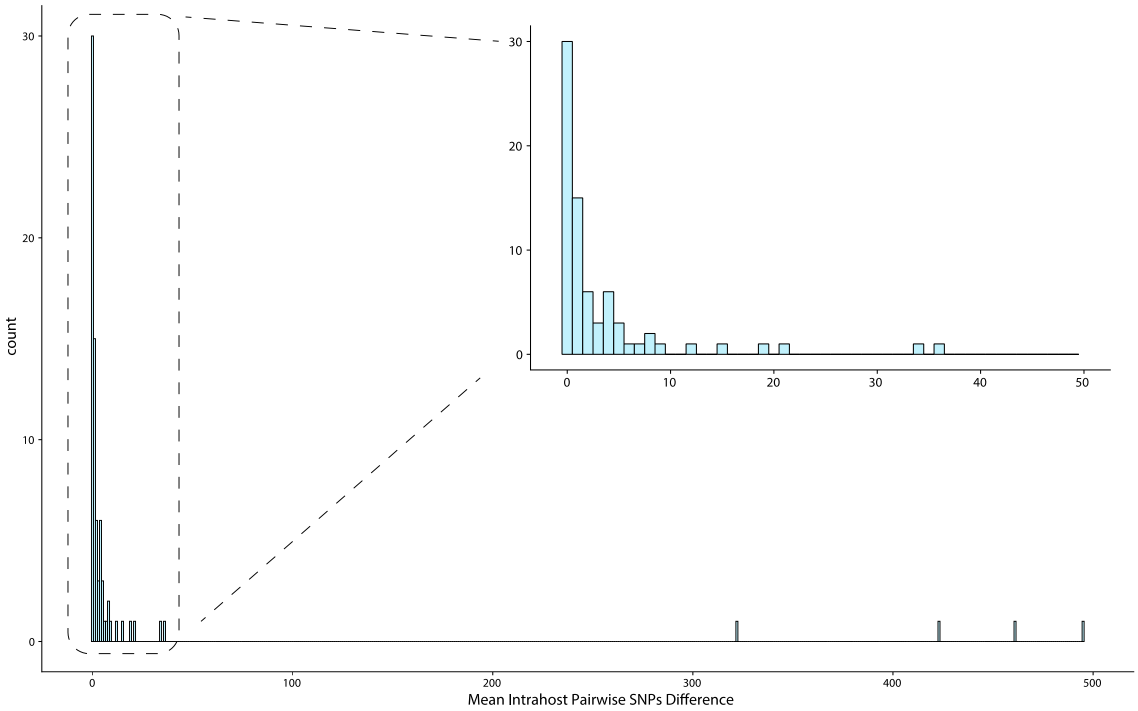

Supplemental Figure 2. Mean pairwise SNP-distances among intrahost individual SA ISLs with ³2 isolates of the same MLST (n=78). An enlarged view of pairwise distances in the 0-50 SNP region of interest is shown on the top right of the figure.

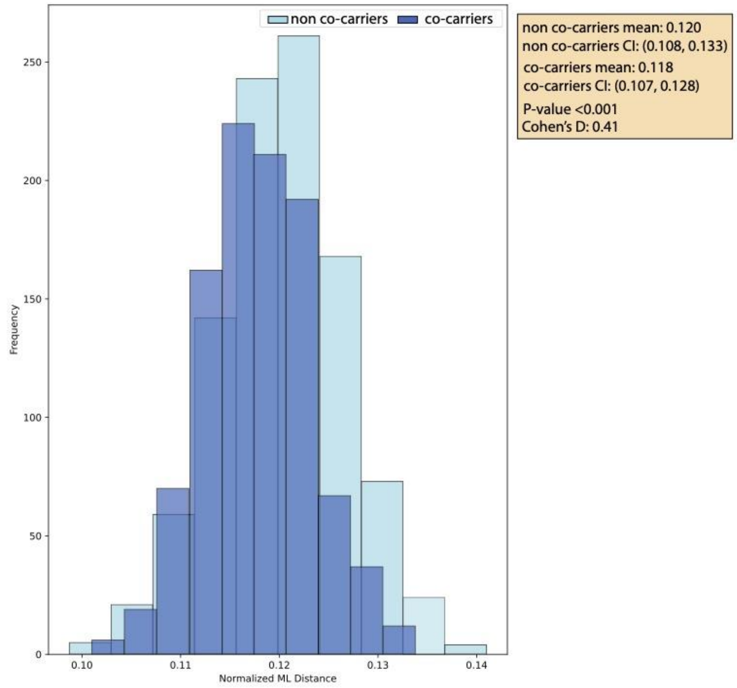

Supplemental Figure 3. Results of permutation test assessing the maximum likelihood (ML) distances among co-carried from the same individual and non-co-carried strains from all individuals. Pairwise ML distances were subsampled to obtain 200 pairs of isolates equally balanced between co-carried and non-co-carried strains for 1000 iterations. Empirical p-value results are for the Mann-Whitney U test comparing the two distributions of mean distances. The Cohen's D value represents the effect size defined as small d=0.2, medium d=0.5, and large d=0.8.

Supplemental Table 1. Individual characteristics of study participants and correlates of co-carriage among Indigenous adults in the Southwest US in 2017.

		Total population (N=60) n (%)	Co-carrier (n=25) n (%)	p-value[a]	Crude prevalence ratio (95% CI)	Age-adjusted prevalence ratio (95% CI)
Age group (years)	18-39	23 (38.3)	13 (56.5)	0.21	Ref	-
	40-64	23 (38.3)	7 (30.4)		0.54 (0.26, 1.10)	-
	≥65	14 (23.3)	5 (35.7)		0.63 (0.29, 1.39)	-
Sex	Male	12 (20.0)	7 (58.3)	0.21	1.56 (0.85, 2.84)	1.37 (0.79, 2.39)
	Female	48 (80.0)	18 (37.5)		Ref	Ref
Type of living facility	Residence	60 (100.0)	25 (41.7)	-	Ref	Ref
	Currently homeless	0	0		-	-
Times likely to bathe or shower in a typical week	1-2	9 (15.3)	3 (33.3)	0.39	Ref	Ref
	3-4	18 (30.5)	10 (55.6)		1.67 (0.61, 4.59)	1.47 (0.50, 4.35)
	5-6	6 (10.2)	1 (16.7)		0.50 (0.07, 3.75)	0.43 (0.05, 3.79)
	7+	26 (44.1)	11 (42.3)		1.27 (0.45, 3.55)	1.16 (0.39, 3.42)
Current tobacco smoker	No	51 (85.0)	21 (41.2)	1.00	Ref	Ref
	Yes	9 (15.0)	4 (44.4)		1.08 (0.48, 2.40)	0.91 (0.44, 1.87)
Current chewing tobacco user	No	55 (91.7)	24 (43.6)	0.39	Ref	Ref
	Yes	5 (8.3)	1 (20.0)		0.46 (0.08, 2.71)	0.44 (0.08, 2.33)
Self-reported history of skin infections or complications (e.g. boils, spider bites, eczema)	No	49 (81.7)	20 (40.8)	1.00	Ref	Ref
	Yes	11 (18.3)	5 (45.5)		1.11 (0.54, 2.31)	0.97 (0.48, 1.99)
Documented SA infection in the past 5 years	No	58 (96.7)	24 (41.4)	1.00	Ref	Ref
	Yes	2 (3.3)	1 (50.0)		1.21 (0.29, 5.00)	1.75 (0.37, 8.18)
Antibiotic use in the past 14 days	No	56 (96.6)	24 (42.9)	0.51	Ref	Ref
	Yes	2 (3.5)	0		-	-
Any underlying health condition	No	21 (35.0)	12 (57.1)	0.10	Ref	Ref
	Yes	39 (65.0)	13 (33.3)		0.58 (0.33, 1.04)	0.65 (0.36, 1.16)
Diabetes	No	42 (70.0)	19 (45.2)	0.57	Ref	Ref
	Yes	18 (30.0)	6 (33.3)		0.74 (0.35, 1.53)	0.98 (0.43, 2.25)
Obesity	No	28 (46.7)	13 (46.4)	0.48	Ref	Ref
	Yes	32 (53.3)	12 (37.5)		0.81 (0.44, 1.47)	0.86 (0.48, 1.54)
Behaviors in the past 6 months						
Hospitalization	No	51 (85.0)	18 (35.3)	0.03	Ref	Ref
	Yes	9 (15.0)	7 (77.8)		**2.20 (1.32, 3.67)**	**2.13 (1.20, 3.77)**
Surgery	No	52 (86.7)	20 (38.5)	0.26	Ref	Ref
	Yes	8 (13.3)	5 (62.5)		1.63 (0.86, 3.07)	1.58 (0.91, 2.76)
Shared a towel	No	46 (76.7)	20 (43.5)	0.76	Ref	Ref
	Yes	14 (23.3)	5 (35.7)		0.82 (0.38, 1.79)	0.97 (0.42, 2.27)
Shared a bed	No	35 (58.3)	13 (37.1)	0.40	Ref	Ref
	Yes	25 (41.7)	12 (48.0)		1.29 (0.71, 2.34)	1.29 (0.71, 2.34)
Worn clothes more than once without washing	No	39 (65.0)	17 (43.6)	0.79	Ref	Ref
	Yes	21 (35.0)	21 (35.0)		0.87 (0.46, 1.68)	0.88 (0.47, 1.65)
Shared clothes	No	59 (98.3)	25 (42.4)	1.00	Ref	Ref

	Yes	1 (1.7)	0		-	-
Gym use	No	51 (85.0)	19 (37.3)	0.15	Ref	Ref
	Yes	9 (15.0)	6 (66.7)		**1.79 (1.00, 3.21)**	**1.84 (1.07, 3.17)**
Used a locker room	No	56 (93.3)	23 (41.1)	1.00	Ref	Ref
	Yes	4 (6.7)	2 (50.0)		1.22 (0.44, 3.41)	1.00 (0.39, 2.54)
Went to a sweat lodge	No	59 (98.3)	24 (40.7)	0.42	Ref	Ref
	Yes	1 (1.7)	1 (100.0)		-	-
Participated in organized team sports or activities	No	56 (93.3)	22 (39.3)	0.30	Ref	Ref
	Yes	4 (6.7)	3 (75.0)		**1.96 (1.02, 3.79)**	1.64 (0.98, 2.75)
Received any artistic (body-art) or cosmetic tattoos	No	55 (91.7)	23 (41.8)	1.00	Ref	Ref
	Yes	5 (8.3)	2 (40.0)		0.98 (0.32, 3.01)	0.88 (0.33, 2.31)
Received any body or facial piercings	No	57 (95.0)	24 (42.1)	1.00	Ref	Ref
	Yes	3 (5.0)	1 (33.3)		0.81 (0.16, 4.15)	0.70 (0.16, 3.09)
Used drugs for non-medical purposes	No	57 (95.0)	25 (43.9)	0.26	Ref	Ref
	Yes	3 (5.0)	0		-	-

[a] *p-value from Chi-square test or Fisher's exact test, as appropriate*

Bold indicates p<0.05 from Poisson regression with robust variance estimation

Supplemental Table 2. Household characteristics of study participants and correlates of co-carriage among Indigenous adults in the Southwest US in 2017.

		**Total population (N=60) n (%)**	**Co-carrier (n=25) n (%)**	**p-value[a]**	**Crude prevalence ratio (95% CI)**	**Age-adjusted prevalence ratio (95% CI)**
Number of people in the household, n (%)	1-2	20 (33.3)	7 (35.0)	0.02	Ref	Ref
	3-4	23 (38.3)	6 (26.1)		0.75 (0.30, 1.85)	0.69 (0.28, 1.68)
	5-6	14 (23.3)	9 (64.3)		1.84 (0.90, 3.75)	1.51 (0.71, 3.23)
	7+	3 (5.0)	3 (100.0)		**2.86 (1.57, 5.19)**	**2.69 (1.45, 4.99)**
Any children in the household	No	45 (75.0)	18 (40.0)	0.76	Ref	Ref
	Yes	15 (25.0)	7 (46.7)		1.12 (0.61, 2.23)	0.96 (0.48, 1.92)
Smoker in the household, n (%)	No	56 (94.9)	22 (39.3)	0.56	Ref	Ref
	Yes	3 (5.1)	2 (66.7)		1.70 (0.72, 4.03)	1.74 (0.75, 4.04)
Number of rooms in the house, n (%)	1-2	7 (11.7)	2 (28.6)	0.66	Ref	Ref
	3-4	18 (30.0)	9 (50.0)		1.75 (0.50, 6.16)	1.66 (0.44, 6.34)
	5-6	18 (30.0)	6 (33.3)		1.12 (0.31, 4.46)	1.21 (0.29, 5.15)
	7+	17 (28.3)	8 (47.1)		1.65 (0.46, 5.90)	1.53 (0.40, 5.82)
Number of rooms for sleeping in the house	1-2	35 (58.3)	14 (40.0)	0.76	Ref	Ref
	3+	25 (41.7)	11 (44.0)		1.10 (0.60, 2.00)	1.06 (0.59, 1.92)
People per bedroom, n (%)	£ 2	53 (88.3)	20 (37.7)	0.12	Ref	Ref
	>2	7 (11.7)	5 (71.4)		**1.89 (1.06, 3.39)**	1.71 (0.87, 3.35)
Presence of indoor piped water	No	14 (23.3)	5 (35.7)	0.76	Ref	Ref
	Yes	46 (76.7)	20 (43.5)		1.22 (0.56, 2.65)	1.01 (0.45, 2.26)
Use of a wood-burning stove	No	51 (85.0)	22 (43.1)	0.72	Ref	Ref
	Yes	9 (15.0)	3 (33.3)		0.77 (0.29, 2.05)	0.87 (0.31, 2.49)
Presence of pet in the house	No	41 (68.3)	17 (41.5)	1.00	Ref	Ref
	Yes	19 (31.7)	8 (42.1)		1.02 (0.54, 1.93)	1.10 (0.60, 2.03)
Presence of livestock	No	44 (73.3)	17 (38.6)	0.35	Ref	Ref
	Yes	14 (23.3)	8 (57.1)		1.08 (0.63, 1.83)	1.15 (0.66, 2.01)
Self-reported history of skin infections or complications among household members (e.g. boils, spider bites, eczema)	No	53 (88.3)	23 (43.4)	0.69	Ref	Ref
	Yes	7 (11.7)	2 (28.6)		0.66 (0.20, 2.21)	0.70 (0.21, 2.29)
Self-reported history of *S. aureus* infection among household members	No	53 (93.0)	20 (37.7)	0.64	Ref	Ref
	Yes	4 (7.0)	2 (50.0)		1.33 (0.47, 3.75)	1.23 (0.41, 3.67)
Household member with chronic illness requiring frequent healthcare	No	45 (75.0)	17 (37.8)	0.37	Ref	Ref
	Yes	15 (25.0)	8 (53.3)		1.41 (0.77, 2.58)	1.70 (0.97, 2.99)
Behaviors of household members in the past 6 months						
Hospitalization	No	54 (90.0)	24 (44.4)	0.39	Ref	Ref
	Yes	6 (10.0)	1 (16.7)		0.38 (0.06, 2.30)	0.35 (0.05, 2.42)
Surgery	No	59 (98.3)	25 (42.4)	1.00	Ref	Ref
	Yes	1 (1.7)	0		-	-
Long-term care residence	No	59 (98.3)	25 (42.4)	1.00	Ref	Ref
	Yes	1 (1.7)	0		-	-
Shared a towel	No	47 (78.3)	22 (46.8)	0.20	Ref	Ref
	Yes	13 (21.7)	3 (23.1)		0.49 (0.17, 1.39)	0.49 (0.17, 1.37)
Shared a bed	No	34 (56.7)	14 (41.2)	1.00	Ref	Ref

	Yes	26 (43.3)	11 (42.3)		1.03 (0.56, 1.88)	0.79 (0.41, 1.50)
Worn clothes more than once without washing	No	51 (85.0)	20 (39.2)	0.47	Ref	Ref
	Yes	9 (15.0)	5 (55.6)		1.42 (0.72, 2.79)	1.34 (0.66, 2.73)
Shared clothes	No	58 (96.7)	25 (43.1)	0.51	Ref	Ref
	Yes	2 (3.3)	0		-	-
Used a gym	No	53 (88.3)	22 (41.5)	1.00	Ref	Ref
	Yes	7 (11.7)	3 (42.9)		1.03 (0.41, 2.57)	1.16 (0.52, 2.59)
Used a locker room	No	54 (90.0)	21 (38.9)	0.22	Ref	Ref
	Yes	6 (10.0)	4 (66.7)		1.71 (0.89, 3.31)	1.50 (0.87, 2.58)
Went to a sweat lodge	No	59 (98.3)	24 (40.7)	0.42	Ref	Ref
	Yes	1 (1.7)	1 (100.0)		2.46 (1.81, 3.35)	1.83 (1.25, 2.68)

[a] *p-value from Chi-square test or Fisher's exact test, as appropriate*

Bold indicates p<0.05 from Poisson regression with robust variance estimation

Supplemental File 1. Accession numbers

Isolate	ISL	MLST	Growth	IR	Biosample accession	Reads accession
AN_005_G1	1321	51	Heavy	Y	SAMN15567908	SRR12343931 (S); SRR12343857 (L)
AN_005_G2	1321	51	Heavy	N	SAMN35040330	SRR24507825
AN_005_G3	1321	51	Heavy	N	SAMN35040331	SRR24507858
NP_005_G1	1321	51	Intermediate	N	SAMN35040406	SRR24507741
NP_005_G2	1321	51	Intermediate	N	SAMN35040407	SRR24507740
NP_005_G3	1321	51	Intermediate	N	SAMN35040408	SRR24507738
OP_007_G1	1333	188	Heavy	Y	SAMN15567976	SRR12343931 (S); SRR12343857 (L)
OP_007_G2	1333	188	Heavy	N	SAMN35040489	SRR24507935
OP_007_G3	1333	188	Heavy	N	SAMN35040490	SRR24507934
OP_007_G4	1333	188	Heavy	N	SAMN35040491	SRR24507933
OP_007_G5	1333	188	Heavy	N	SAMN35040492	SRR24507932
OP_007_G6	1333	188	Heavy	N	SAMN35040493	SRR24507931
AN_016_G1	1341	97	Heavy	Y	SAMN15567910	SRR12343931 (S); SRR12343857 (L)
AN_016_G2	1341	97	Heavy	N	SAMN35040332	SRR24507847
AN_016_G3	1341	97	Heavy	N	SAMN35040333	SRR24507804
AN_016_G4	1341	97	Heavy	N	SAMN35040334	SRR24507793
AN_023_G1	1345	72	Light	Y	SAMN15567911	SRR12343931 (S); SRR12343857 (L)
AN_023_G2	1345	72	Light	N	SAMN35040335	SRR24507782
AN_023_G3	1345	72	Light	N	SAMN35040336	SRR24507739
NP_022_G1	1345	72	Intermediate	N	SAMN35040409	SRR24507737
NP_022_G2	1345	72	Intermediate	N	SAMN35040410	SRR24507736
NP_022_G3	1345	72	Intermediate	N	SAMN35040411	SRR24507735
AN_036_G1	1365	188	Heavy	Y	SAMN15567913	SRR12343931 (S); SRR12343857 (L)
AN_036_G2	1365	188	Heavy	N	SAMN35040337	SRR24507728
AN_036_G3	1365	188	Heavy	N	SAMN35040338	SRR24507903
AN_036_G4	1365	188	Heavy	N	SAMN35040339	SRR24507960
AN_050_G1	1370	45	Intermediate	Y	SAMN15567916	SRR12343931 (S); SRR12343857 (L)
AN_050_G2	1370	45	Intermediate	N	SAMN35040340	SRR24507949
AN_050_G3	1370	45	Intermediate	N	SAMN35040341	SRR24507938
NP_049_G1	1370	45	Light	N	SAMN35040412	SRR24507734
NP_049_G2	1370	45	Light	N	SAMN35040413	SRR24507733
NP_049_G3	1370	45	Light	N	SAMN35040414	SRR24507732
OP_035_G1	1386	188	Heavy	Y	SAMN15567979	SRR12343931 (S); SRR12343857 (L)
OP_035_G2	1386	188	Heavy	N	SAMN35040498	SRR24507925
OP_035_G3	1386	188	Heavy	N	SAMN35040499	SRR24507924
OP_035_G4	1386	188	Heavy	N	SAMN35040500	SRR24507923
OP_026_G1	1387	72	Heavy	Y	SAMN15567978	SRR12343931 (S); SRR12343857 (L)
OP_026_G2	1387	72	Heavy	N	SAMN35040494	SRR24507930
OP_026_G3	1387	72	Heavy	N	SAMN35040495	SRR24507929
OP_026_G4	1387	72	Heavy	N	SAMN35040496	SRR24507928
OP_043_G1	3402	45	Heavy	Y	SAMN15567981	SRR12343931 (S); SRR12343857 (L)
OP_043_G2	3402	45	Heavy	N	SAMN35040504	SRR24507919
OP_043_G3	3402	45	Heavy	N	SAMN35040505	SRR24507918
OP_043_G4	3402	45	Heavy	N	SAMN35040506	SRR24507917
AN_079_G1	3406	5	Heavy	Y	SAMN15567920	SRR12343931 (S); SRR12343857 (L)
AN_079_G2	3406	5	Heavy	N	SAMN35040347	SRR24507836
AN_079_G3	3406	5	Heavy	N	SAMN35040348	SRR24507824
AN_079_G4	3406	5	Heavy	N	SAMN35040349	SRR24507813
OP_042_G1	3417	39	Heavy	Y	SAMN15567980	SRR12343931 (S); SRR12343857 (L)
OP_042_G2	3417	39	Heavy	N	SAMN35040501	SRR24507922
OP_042_G3	3417	39	Heavy	N	SAMN35040502	SRR24507921
OP_042_G4	3417	39	Heavy	N	SAMN35040503	SRR24507920

OP_052_G1	3422	188	Heavy	Y	SAMN15567982	SRR12343931 (S); SRR12343857 (L)
OP_052_G2	3422	188	Heavy	N	SAMN35040507	SRR24507916
OP_052_G3	3422	188	Heavy	N	SAMN35040508	SRR24507914
OP_052_G4	3422	188	Heavy	N	SAMN35040509	SRR24507913
OP_052_G5	3422	188	Heavy	N	SAMN35040510	SRR24507912
OP_052_G6	3422	188	Heavy	N	SAMN35040511	SRR24507911
OP_056_G1	3426	72	Heavy	Y	SAMN15567983	SRR12343931 (S); SRR12343857 (L)
OP_056_G2	3426	72	Heavy	N	SAMN35040512	SRR24507910
OP_056_G3	3426	72	Heavy	N	SAMN35040513	SRR24507909
OP_056_G4	3426	72	Heavy	N	SAMN35040514	SRR24507908
AN_111_G1	3442	5	Heavy	Y	SAMN15567928	SRR12343931 (S); SRR12343857 (L)
AN_111_G2	3442	5	Heavy	N	SAMN35040354	SRR24507862
AN_111_G3	3442	5	Heavy	N	SAMN35040355	SRR24507861
AN_111_G4	3442	5	Heavy	N	SAMN35040356	SRR24507860
AN_123_G1	3460	8	Heavy	Y	SAMN15567930	SRR12343931 (S); SRR12343857 (L)
AN_123_G2	3460	8	Heavy	N	SAMN35040359	SRR24507856
AN_123_G3	3460	8	Heavy	N	SAMN35040360	SRR24507855
NP_122_G1	3460	8	Heavy	N	SAMN35040428	SRR24507902
NP_122_G2	3460	8	Heavy	N	SAMN35040429	SRR24507969
NP_122_G3	3460	8	Heavy	N	SAMN35040430	SRR24507968
OP_070_G1	3460	8	Light	N	SAMN35040520	SRR24507833
OP_070_G2	3460	8	Light	N	SAMN35040521	SRR24507832
OP_070_G3	3460	8	Light	N	SAMN35040522	SRR24507831
OP_071_G1	3471	188	Heavy	Y	SAMN15567985	SRR12343931 (S); SRR12343857 (L)
OP_071_G2	3471	188	Heavy	N	SAMN35040523	SRR24507830
OP_071_G3	3471	188	Heavy	N	SAMN35040524	SRR24507829
OP_071_G4	3471	188	Heavy	N	SAMN35040525	SRR24507828
AN_130_G1	4555	59	Intermediate	Y	SAMN15567931	SRR12343931 (S); SRR12343857 (L)
AN_130_G2	4555	59	Intermediate	N	SAMN35040361	SRR24507854
AN_130_G3	4555	59	Intermediate	N	SAMN35040362	SRR24507853
NP_129_G1	4555	59	Light	N	SAMN35040431	SRR24507967
AN_136_G1	4561	97	Heavy	Y	SAMN15567932	SRR12343931 (S); SRR12343857 (L)
AN_136_G2	4561	97	Heavy	N	SAMN35040363	SRR24507852
AN_136_G3	4561	97	Heavy	N	SAMN35040364	SRR24507851
NP_135_G1	4561	97	Heavy	N	SAMN35040432	SRR24507966
NP_135_G2	4561	97	Heavy	N	SAMN35040433	SRR24507965
NP_135_G3	4561	97	Heavy	N	SAMN35040434	SRR24507964
AN_141_G1	4566	30	Heavy	Y	SAMN15567933	SRR12343931 (S); SRR12343857 (L)
AN_141_G2	4566	30	Heavy	N	SAMN35040365	SRR24507850
AN_141_G3	4566	30	Heavy	N	SAMN35040366	SRR24507849
AN_141_G4	4566	30	Heavy	N	SAMN35040367	SRR24507848
AN_142_G1	4567	7314	Heavy	Y	SAMN15567934	SRR12343931 (S); SRR12343857 (L)
OP_081_G1	4567	7314	Heavy	N	SAMN35040526	SRR24507827
OP_081_G2	4567	7314	Heavy	N	SAMN35040527	SRR24507826
OP_081_G3	4567	7314	Heavy	N	SAMN35040528	SRR24507823
OP_087_G1	4580	5	Heavy	Y	SAMN15567986	SRR12343931 (S); SRR12343857 (L)
OP_087_G2	4580	5	Heavy	N	SAMN35040535	SRR24507816
OP_087_G3	4580	5	Heavy	N	SAMN35040536	SRR24507815
OP_087_G4	4580	5	Heavy	N	SAMN35040537	SRR24507814
OP_101_G1	4606	8	Heavy	Y	SAMN15567988	SRR12343931 (S); SRR12343857 (L)
OP_101_G2	4606	8	Heavy	N	SAMN35040542	SRR24507808
OP_101_G3	4606	8	Heavy	N	SAMN35040543	SRR24507807
OP_101_G4	4606	8	Heavy	N	SAMN35040544	SRR24507806
OP_107_G1	4614	97	Heavy	Y	SAMN15567989	SRR12343931 (S); SRR12343857 (L)
OP_107_G2	4614	97	Heavy	N	SAMN35040545	SRR24507773

OP_107_G3	4614	97	Heavy	N	SAMN35040546	SRR24507772
OP_107_G4	4614	97	Heavy	N	SAMN35040547	SRR24507771
NP_198_G1	7280	5	Light	Y	SAMN15567970	SRR12343931 (S); SRR12343857 (L)
NP_198_G2	7280	5	Light	N	SAMN35040444	SRR24507953
NP_198_G3	7280	5	Light	N	SAMN35040445	SRR24507952
NP_198_G4	7280	5	Light	N	SAMN35040446	SRR24507951
NP_198_G5	7280	5	Light	N	SAMN35040447	SRR24507950
OP_132_G1	7287	188	Heavy	Y	SAMN15567992	SRR12343931 (S); SRR12343857 (L)
OP_132_G2	7287	188	Heavy	N	SAMN35040554	SRR24507763
OP_132_G3	7287	188	Heavy	N	SAMN35040555	SRR24507762
OP_132_G4	7287	188	Heavy	N	SAMN35040556	SRR24507761
NP_223_G1	7288	7317	Light	Y	SAMN15567973	SRR12343931 (S); SRR12343857 (L)
NP_223_G2	7288	7317	Light	N	SAMN35040463	SRR24507896
NP_223_G3	7288	7317	Light	N	SAMN35040464	SRR24507895
NP_223_G4	7288	7317	Light	N	SAMN35040465	SRR24507894
NP_223_G5	7288	7317	Light	N	SAMN35040466	SRR24507893
NP_223_G6	7288	7317	Light	N	SAMN35040467	SRR24507892
OP_133_G1	7292	8	Heavy	Y	SAMN15567993	SRR12343931 (S); SRR12343857 (L)
OP_133_G2	7292	8	Heavy	N	SAMN35040557	SRR24507760
OP_133_G3	7292	8	Heavy	N	SAMN35040558	SRR24507758
OP_133_G4	7292	8	Heavy	N	SAMN35040559	SRR24507757
AN_204_G1	7402	5	Heavy	Y	SAMN15567945	SRR12343931 (S); SRR12343857 (L)
AN_204_G2	7402	5	Heavy	N	SAMN35040374	SRR24507840
AN_204_G3	7402	5	Heavy	N	SAMN35040375	SRR24507839
AN_204_G4	7402	5	Heavy	N	SAMN35040376	SRR24507838
AN_206_G1	7404	188	Heavy	Y	SAMN15567947	SRR12343931 (S); SRR12343857 (L)
AN_206_G2	7404	188	Heavy	N	SAMN35040379	SRR24507802
AN_206_G3	7404	188	Heavy	N	SAMN35040380	SRR24507801
NP_205_G1	7404	188	Light	N	SAMN35040454	SRR24507942
NP_205_G2	7404	188	Light	N	SAMN35040455	SRR24507941
NP_205_G3	7404	188	Light	N	SAMN35040456	SRR24507940
OP_120_G1	7404	188	Heavy	N	SAMN35040551	SRR24507766
OP_120_G2	7404	188	Heavy	N	SAMN35040552	SRR24507765
OP_120_G3	7404	188	Heavy	N	SAMN35040553	SRR24507764
NP_215_G1	7413	7317	Intermediate	Y	SAMN15567972	SRR12343931 (S); SRR12343857 (L)
NP_215_G2	7413	7317	Intermediate	N	SAMN35040457	SRR24507939
NP_215_G3	7413	7317	Intermediate	N	SAMN35040458	SRR24507901
NP_215_G4	7413	7317	Intermediate	N	SAMN35040459	SRR24507900
OP_129_G1	7415	72	Heavy	Y	SAMN15567990	SRR12343931 (S); SRR12343857 (L)
NP_219_G1	7415	72	Intermediate	N	SAMN35040460	SRR24507899
NP_219_G2	7415	72	Intermediate	N	SAMN35040461	SRR24507898
NP_219_G3	7415	72	Intermediate	N	SAMN35040462	SRR24507897
AN_247_G1	7436	72	Heavy	Y	SAMN15567952	SRR12343931 (S); SRR12343857 (L)
AN_247_G2	7436	72	Heavy	N	SAMN35040387	SRR24507794
AN_247_G3	7436	72	Heavy	N	SAMN35040388	SRR24507792
AN_247_G4	7436	72	Heavy	N	SAMN35040389	SRR24507791
AN_265_G1	9372	25	Heavy	Y	SAMN15567957	SRR12343931 (S); SRR12343857 (L)
AN_265_G2	9372	25	Heavy	N	SAMN35040397	SRR24507783
AN_265_G3	9372	25	Heavy	N	SAMN35040398	SRR24507781
NP_264_G1	9372	25	Heavy	N	SAMN35040479	SRR24507878
NP_264_G2	9372	25	Heavy	N	SAMN35040480	SRR24507877
NP_264_G3	9372	25	Heavy	N	SAMN35040481	SRR24507876
OP_163_G1	9372	25	Light	N	SAMN15567999	SRR12343859 (S); SRR12343942(L)
OP_163_G2	9372	25	Light	N	SAMN35040577	SRR24507866
OP_163_G3	9372	25	Light	N	SAMN35040578	SRR24507864

OP_154_G1	9435	5	Heavy	Y	SAMN15567998	SRR12343931 (S); SRR12343857 (L)
OP_154_G2	9435	5	Heavy	N	SAMN35040573	SRR24507742
OP_154_G3	9435	5	Heavy	N	SAMN35040574	SRR24507869
OP_154_G4	9435	5	Heavy	N	SAMN35040575	SRR24507868
OP_153_G1	9439	8	Heavy	Y	SAMN15567997	SRR12343931 (S); SRR12343857 (L)
OP_153_G2	9439	8	Heavy	N	SAMN35040569	SRR24507746
OP_153_G3	9439	8	Heavy	N	SAMN35040570	SRR24507745
OP_153_G4	9439	8	Heavy	N	SAMN35040571	SRR24507744
OP_153_G5	9439	8	Heavy	N	SAMN35040572	SRR24507743
AN_003_G1	1320_188	188	Heavy	Y	SAMN15567907	SRR12343931 (S); SRR12343857 (L)
AN_003_G2	1320_188	188	Heavy	N	SAMN35040328	SRR24507905
AN_003_G3	1320_188	188	Heavy	N	SAMN35040329	SRR24507904
NP_003_G1	1320_188	188	Intermediate	N	SAMN35040403	SRR24507776
NP_003_G2	1320_188	188	Intermediate	N	SAMN35040404	SRR24507775
NP_003_G3	1320_188	188	Intermediate	N	SAMN35040405	SRR24507774
OP_003_G1	1320_7317	7317	Light	N	SAMN35040488	SRR24507936
AN_053_G1	1376_30	30	Heavy	Y	SAMN35040342	SRR24507891 (S); SRR24507863 (L)
AN_053_G2	1376_30	30	Heavy	N	SAMN35040343	SRR24507880
AN_053_G3	1376_30	30	Heavy	N	SAMN35040344	SRR24507937
NP_052_G1	1376_30	30	Light	N	SAMN35040415	SRR24507731
NP_052_G2	1376_30	30	Light	N	SAMN35040416	SRR24507730
NP_052_G3	1376_30	30	Light	N	SAMN35040417	SRR24507729
OP_030_G1	1376_72	72	Light	N	SAMN35040497	SRR24507927
NP_067_G1	3411_6	6	Light	N	SAMN35040418	SRR24507727
NP_067_G2	3411_6	6	Light	N	SAMN35040419	SRR24507726
AN_068_G1	3411_6956	6956	Heavy	Y	SAMN15567918	SRR12343931 (S); SRR12343857 (L)
AN_068_G2	3411_6956	6956	Heavy	N	SAMN35040345	SRR24507926
AN_068_G3	3411_6956	6956	Heavy	N	SAMN35040346	SRR24507915
AN_099_G1	3443_6	6	Heavy	Y	SAMN15567923	SRR12343931 (S); SRR12343857 (L)
AN_099_G2	3443_6	6	Heavy	N	SAMN35040350	SRR24507770
AN_099_G3	3443_6	6	Heavy	N	SAMN35040351	SRR24507759
NP_098_G3	3443_6	6	Intermediate	N	SAMN35040422	SRR24507723
NP_098_G1	3443_6956	6956	Intermediate	N	SAMN35040420	SRR24507725
NP_098_G2	3443_6956	6956	Intermediate	N	SAMN35040421	SRR24507724
AN_115_G1	3448_5	5	Heavy	Y	SAMN15567929	SRR12343931 (S); SRR12343857 (L)
AN_115_G2	3448_5	5	Heavy	N	SAMN35040357	SRR24507859
AN_115_G3	3448_5	5	Heavy	N	SAMN35040358	SRR24507857
NP_114_G1	3448_5	5	Intermediate	N	SAMN35040425	SRR24507720
NP_114_G2	3448_5	5	Intermediate	N	SAMN35040426	SRR24507719
NP_114_G3	3448_5	5	Intermediate	N	SAMN35040427	SRR24507718
OP_066_G1	3448_72	72	Light	N	SAMN35040517	SRR24507837
OP_066_G2	3448_72	72	Light	N	SAMN35040518	SRR24507835
OP_066_G3	3448_72	72	Light	N	SAMN35040519	SRR24507834
NP_107_G1	3453_188	188	Light	N	SAMN35040423	SRR24507722
NP_107_G2	3453_188	188	Light	N	SAMN35040424	SRR24507721
OP_063_G1	3453_8	8	Light	Y	SAMN15567984	SRR12343931 (S); SRR12343857 (L)
OP_063_G2	3453_8	8	Light	N	SAMN35040515	SRR24507907
OP_064_G1	3455_5	5	Light	N	SAMN35040516	SRR24507906
AN_109_G1	3455_72	72	Light	Y	SAMN15567927	SRR12343931 (S); SRR12343857 (L)
AN_109_G2	3455_72	72	Light	N	SAMN35040352	SRR24507748
AN_109_G3	3455_72	72	Light	N	SAMN35040353	SRR24507865
OP_082_G1	4568_15	15	Light	N	SAMN35040529	SRR24507822
OP_082_G2	4568_15	15	Light	N	SAMN35040530	SRR24507821
OP_082_G3	4568_188	188	Light	N	SAMN35040531	SRR24507820
AN_143_G1	4568_5	5	Light	Y	SAMN15567935	SRR12343931 (S); SRR12343857 (L)

AN_143_G2	4568_5	5	Light	N	SAMN35040368	SRR24507846
AN_143_G3	4568_5	5	Light	N	SAMN35040369	SRR24507845
OP_085_G1	4577_1	1	Light	N	SAMN35040532	SRR24507819
OP_085_G2	4577_1	1	Light	N	SAMN35040533	SRR24507818
OP_085_G3	4577_1	1	Light	N	SAMN35040534	SRR24507817
AN_150_G1	4577_30	30	Heavy	Y	SAMN15567937	SRR12343931 (S); SRR12343857 (L)
AN_150_G2	4577_30	30	Heavy	N	SAMN35040370	SRR24507844
AN_150_G3	4577_30	30	Heavy	N	SAMN35040371	SRR24507843
NP_150_G1	4577_30	30	Light	N	SAMN35040435	SRR24507963
NP_150_G2	4577_30	30	Light	N	SAMN35040436	SRR24507962
NP_150_G3	4577_30	30	Light	N	SAMN35040437	SRR24507961
OP_098_G1	4597_5	5	Heavy	Y	SAMN15567987	SRR12343931 (S); SRR12343857 (L)
OP_098_G2	4597_5	5	Heavy	N	SAMN35040538	SRR24507812
OP_098_G3	4597_5	5	Heavy	N	SAMN35040539	SRR24507811
OP_098_G5	4597_5	5	Heavy	N	SAMN35040541	SRR24507809
OP_098_G4	4597_7550	7550	Heavy	N	SAMN35040540	SRR24507810
AN_176_G1	4602_5	5	Light	Y	SAMN15567941	SRR12343931 (S); SRR12343857 (L)
NP_175_G1	4602_7317	7317	Intermediate	N	SAMN35040438	SRR24507959
NP_175_G2	4602_7317	7317	Intermediate	N	SAMN35040439	SRR24507958
NP_175_G3	4602_7317	7317	Intermediate	N	SAMN35040440	SRR24507957
AN_184_G1	4612_5	5	Intermediate	Y	SAMN15567943	SRR12343931 (S); SRR12343857 (L)
NP_183_G2	4612_7317	7317	Intermediate	N	SAMN35040442	SRR24507955
NP_183_G3	4612_7317	7317	Intermediate	N	SAMN35040443	SRR24507954
NP_183_G1	4612_7549	7549	Intermediate	N	SAMN35040441	SRR24507956
OP_111_G1	7276_20	20	Heavy	N	SAMN35040548	SRR24507769
OP_111_G2	7276_20	20	Heavy	N	SAMN35040549	SRR24507768
OP_111_G3	7276_20	20	Heavy	N	SAMN35040550	SRR24507767
NP_196_G1	7276_7317	7317	Light	Y	SAMN15567969	SRR12343931 (S); SRR12343857 (L)
OP_131_G1	7290_5	5	Heavy	Y	SAMN15567991	SRR12343931 (S); SRR12343857 (L)
AN_225_G1	7290_6176	6176	Intermediate	N	SAMN35040381	SRR24507800
AN_225_G2	7290_6176	6176	Intermediate	N	SAMN35040382	SRR24507799
AN_225_G3	7290_6176	6176	Intermediate	N	SAMN35040383	SRR24507798
NP_202_G1	7401_15	15	Intermediate	N	SAMN35040448	SRR24507948
NP_202_G2	7401_188	188	Intermediate	N	SAMN35040449	SRR24507947
NP_202_G3	7401_188	188	Intermediate	N	SAMN35040450	SRR24507946
AN_203_G1	7401_5	5	Intermediate	Y	SAMN15567944	SRR12343931 (S); SRR12343857 (L)
AN_203_G2	7401_5	5	Intermediate	N	SAMN35040372	SRR24507842
AN_203_G3	7401_5	5	Intermediate	N	SAMN35040373	SRR24507841
NP_204_G1	7403_188	188	Intermediate	N	SAMN35040451	SRR24507945
NP_204_G2	7403_30	30	Intermediate	N	SAMN35040452	SRR24507944
AN_205_G1	7403_5	5	Light	Y	SAMN15567946	SRR12343931 (S); SRR12343857 (L)
AN_205_G2	7403_5	5	Light	N	SAMN35040377	SRR24507805
AN_205_G3	7403_5	5	Light	N	SAMN35040378	SRR24507803
NP_204_G3	7403_5	5	Intermediate	N	SAMN35040453	SRR24507943
NP_229_G1	7418_7317	7317	Light	Y	SAMN15567974	SRR12343931 (S); SRR12343857 (L)
NP_229_G2	7418_7317	7317	Light	N	SAMN35040468	SRR24507890
NP_229_G3	7418_7317	7317	Light	N	SAMN35040469	SRR24507889
AN_230_G1	7418_7565	7565	Light	N	SAMN35040384	SRR24507797
OP_138_G1	7427_188	188	Intermediate	N	SAMN35040560	SRR24507756
OP_138_G2	7427_188	188	Intermediate	N	SAMN35040561	SRR24507755
OP_138_G3	7427_188	188	Intermediate	N	SAMN35040562	SRR24507754
AN_239_G1	7427_5	5	Light	Y	SAMN15567951	SRR12343931 (S); SRR12343857 (L)
AN_239_G2	7427_5	5	Light	N	SAMN35040385	SRR24507796
AN_239_G3	7427_5	5	Light	N	SAMN35040386	SRR24507795
OP_143_G1	7434_20	20	Heavy	Y	SAMN15567994	SRR12343931 (S); SRR12343857 (L)

OP_143_G2	7434_34	34	Heavy	N	SAMN35040563	SRR24507753
OP_143_G3	7434_34	34	Heavy	N	SAMN35040564	SRR24507752
OP_143_G4	7434_34	34	Heavy	N	SAMN35040565	SRR24507751
OP_146_G1	7439_6177	6177	Intermediate	N	SAMN15567995	SRR12343863 (S); SRR12343946 (L)
OP_146_G2	7439_6177	6177	Intermediate	N	SAMN35040567	SRR24507749
OP_146_G3	7439_6177	6177	Intermediate	N	SAMN35040568	SRR24507747
AN_250_G1	7439_9	9	Intermediate	Y	SAMN15567953	SRR12343931 (S); SRR12343857 (L)
AN_250_G2	7439_9	9	Intermediate	N	SAMN35040390	SRR24507790
AN_250_G3	7439_9	9	Intermediate	N	SAMN35040391	SRR24507789
NP_249_G1	7439_9	9	Light	N	SAMN35040470	SRR24507888
NP_249_G2	7439_9	9	Light	N	SAMN35040471	SRR24507887
NP_249_G3	7439_9	9	Light	N	SAMN35040472	SRR24507886
OP_147_G1	7440_188	188	Heavy	Y	SAMN15567996	SRR12343931 (S); SRR12343857 (L)
NP_250_G1	7440_25	25	Intermediate	N	SAMN35040473	SRR24507885
NP_250_G2	7440_25	25	Intermediate	N	SAMN35040474	SRR24507884
NP_250_G3	7440_5	5	Intermediate	N	SAMN35040475	SRR24507883
AN_270_G1	9398_188	188	Intermediate	Y	SAMN15567958	SRR12343931 (S); SRR12343857 (L)
AN_270_G2	9398_188	188	Intermediate	N	SAMN35040399	SRR24507780
AN_270_G3	9398_188	188	Intermediate	N	SAMN35040400	SRR24507779
NP_269_G2	9398_5	5	Heavy	N	SAMN35040483	SRR24507874
NP_269_G3	9398_5	5	Heavy	N	SAMN35040484	SRR24507873
NP_269_G1	9398_51	51	Heavy	N	SAMN35040482	SRR24507875
AN_260_G1	9409_1	1	Heavy	Y	SAMN15567955	SRR12343931 (S); SRR12343857 (L)
AN_260_G2	9409_188	188	Heavy	N	SAMN35040392	SRR24507788
AN_260_G3	9409_188	188	Heavy	N	SAMN35040393	SRR24507787
AN_260_G4	9409_188	188	Heavy	N	SAMN35040394	SRR24507786
NP_260_G1	9411_30	30	Heavy	N	SAMN35040476	SRR24507882
AN_261_G1	9411_51	51	Heavy	Y	SAMN15567956	SRR12343931 (S); SRR12343857 (L)
AN_261_G2	9411_51	51	Heavy	N	SAMN35040395	SRR24507785
AN_261_G3	9411_51	51	Heavy	N	SAMN35040396	SRR24507784
NP_260_G2	9411_51	51	Heavy	N	SAMN35040477	SRR24507881
NP_260_G3	9411_51	51	Heavy	N	SAMN35040478	SRR24507879
AN_286_G1	9450_188	188	Intermediate	Y	SAMN15567961	SRR12343931 (S); SRR12343857 (L)
AN_286_G2	9450_188	188	Intermediate	N	SAMN35040401	SRR24507778
AN_286_G3	9450_188	188	Intermediate	N	SAMN35040402	SRR24507777
NP_285_G1	9450_5	5	Intermediate	N	SAMN35040485	SRR24507872
NP_285_G2	9450_5	5	Intermediate	N	SAMN35040486	SRR24507871
NP_285_G3	9450_5	5	Intermediate	N	SAMN35040487	SRR24507870
